# Trojan Horse virus delivering CRISPR-AsCas12f1 controls plant bacterial wilt caused by *Ralstonia solanacearum*

**DOI:** 10.1128/mbio.00619-24

**Published:** 2024-07-16

**Authors:** Shiwen Peng, Yanan Xu, Hao Qu, Fushang Nong, Fangling Shu, Gaoqing Yuan, Lifang Ruan, Dehong Zheng

**Affiliations:** 1 State Key Laboratory for Conservation and Utilization of Subtropical Agro-bioresources, Guangxi Key Laboratory of Agro-environment and Agro-product Safety, College of Agriculture, Guangxi University, Nanning, China; 2 Pharmaceutical College, Guangxi Medical University, Nanning, China; 3 State Key Laboratory of Agricultural Microbiology, College of Life Science and Technology, Huazhong Agricultural University, Wuhan, China; University of Nebraska-Lincoln, Lincoln, Nebraska, USA

**Keywords:** pathogenic bacteria, *Ralstonia solanacearum*, filamentous phage, engineered phage, CRISPR-Cas

## Abstract

Plant bacterial wilt caused by *Ralstonia solanacearum* results in huge losses. Accordingly, developing an effective control method for this disease is urgently required. Filamentous phages, which do not lyse host bacteria and exert minimal burden, offer a potential biocontrol solution. A filamentous phage RSCq that infects *R. solanacearum* was isolated in this study through genome mining. We constructed engineered filamentous phages based on RSCq by employing our proposed approach with wide applicability to non-model phages, enabling the exogenous genes delivery into bacterial cells. CRISPR-AsCas12f1 is a miniature class 2 type V-F CRISPR-Cas system. A CRISPR-AsCas12f1-based gene editing system that targets the key virulence regulator gene *hrpB* was developed, generating the engineered phage RSCqCRISPR-Cas. Similar to the Greek soldiers in the Trojan Horse, our findings demonstrated that the engineered phage-delivered CRISPR-Cas system could disarm the key “weapon,” *hrpB*, of *R. solanacearum*, in medium and plants. Remarkably, pretreatment with RSCqCRISPR-Cas significantly controlled tobacco bacterial wilt, highlighting the potential of engineered filamentous phages as promising biocontrol agents against plant bacterial diseases.

**IMPORTANCE:**

Bacterial disease, one of the major plant diseases, causes huge food and economic losses. Phage therapy, an environmentally friendly control strategy, has been frequently reported in plant bacterial disease control. However, host specificity, sensitivity to ultraviolet light and certain conditions, and bacterial resistance to phage impede the widespread application of phage therapy in crop production. Filamentous phages, which do not lyse host bacteria and exert minimal burden, offer a potential solution to overcome the limitations of lytic phage biocontrol. This study developed a genetic engineering approach with wide applicability to non-model filamentous phages and proved the application possibility of engineered phage-based gene delivery in plant bacterial disease biocontrol for the first.

## INTRODUCTION

An estimated 821 million people—approximately 1 out of every 9 people in the world—are undernourished (1). Moreover, the world’s population is expected to reach 9.6 billion by 2050, and additional global food supply will be needed to meet the increasing demands (2). However, plant diseases cause around 10%–20% of global food production loss every year (3, 4). For instance, the plant bacterial pathogen *Ralstonia solanacearum* species complex infects more than 400 plant species from over 50 families, and losses due to the disease on potatoes alone were estimated at US$1 billion each year worldwide (5). Nevertheless, efficient control methods for managing plant bacterial wilt caused by the *R. solanacearum* species complex remain highly limited.

Efficient control methods for plant diseases are of utmost importance to ensure optimal crop production and meet the ever-increasing demands for food. Among the different strategies, the utilization of phages, which are viruses that infect bacteria, exhibits considerable promise. Phage therapy, an environment-friendly control strategy, has been frequently reported in plant bacterial disease control (6). For example, phage ΦPD10.3 and ΦPD23.1 reduced the severity of potato soft rot caused by *Pectobacterium carotovorum* by 80%–95% (7). In another study, a phage cocktail that consisted of six phages effectively suppressed symptom development of leek bacterial blight caused by *Pseudomonas syringae* (8). Similarly, increasing the number of *R. solanacearum* phages in various combinations decreased the incidence of tomato bacterial wilt disease by up to 80% (9). However, limitations still impede the widespread application of phage therapy in crop production. The host specificity of phages is a major disadvantage that may be partially overcome by the development of phage cocktails. Moreover, the emergence of bacterial resistance to phage infection poses challenges to the continuous use of phage treatments. In addition, phage sensitivity to ultraviolet (UV) light and certain soil conditions cause phage decline after application, undermining the biocontrol effect (6).

In general, strictly lytic phages are preferred for biocontrol applications. However, temperate phages, which are highly abundant, should not be overlooked (10). Filamentous phages belong to the *Inoviridae* family of phages with small single-stranded DNA (ssDNA) genomes packaged within filament-like virions. In contrast with lytic phages, filamentous phages do not lyse or otherwise kill the host bacterium, but instead, egress from the host cell, imposing minimal burden on bacteria (11). *R. solanacearum* loses virulence on tomato plants under the infection of filamentous phages RSM1 and RSM3 (12). Although many other filamentous phages have no biocontrol effect and even enhance the virulence of phytopathogenic bacteria (13), the huge diversity of filamentous prophages integrated into bacterial genomes and the small genome size make filamentous phages promising genetically engineered biocontrol reagents and biotechnological tools (10, 14). Moreover, bacteria infected by filamentous phages continue to produce infectious phage particles, which can potentially counteract the influence of UV light and other hostile environmental conditions. Consequently, filamentous phages offer a potential solution to overcome the limitations of lytic phage biocontrol.

In a nod to the ancient stratagem of the Trojan Horse, where Greek warriors hid inside a colossal wooden equine to infiltrate the fortified city of Troy and ultimately triumph in the Trojan War, our research adopted a comparable approach. We constructed filamentous phage-based “Trojan Horses,” metaphorical “gifts,” aimed at the pathogenic bacteria *R. solanacearum*. Drawing inspiration from the legendary soldiers hidden inside the Trojan Horse, we utilized the clustered regularly interspaced short palindromic repeat and CRISPR-associated proteins (CRISPR-Cas) system, which specifically targets the key virulence regulator gene *hrpB* of *R. solanacearum*. The CRISPR-Cas system was successfully delivered through the engineered phages into *R. solanacearum* cells, leading to efficient control of plant bacterial wilt caused by this pathogen. In essence, our study harnessed the concept of the Trojan Horse to combat this disease effectively.

## RESULTS

### Prophage mining in the genomes of *R. solanacearum*

**Fig 1:**
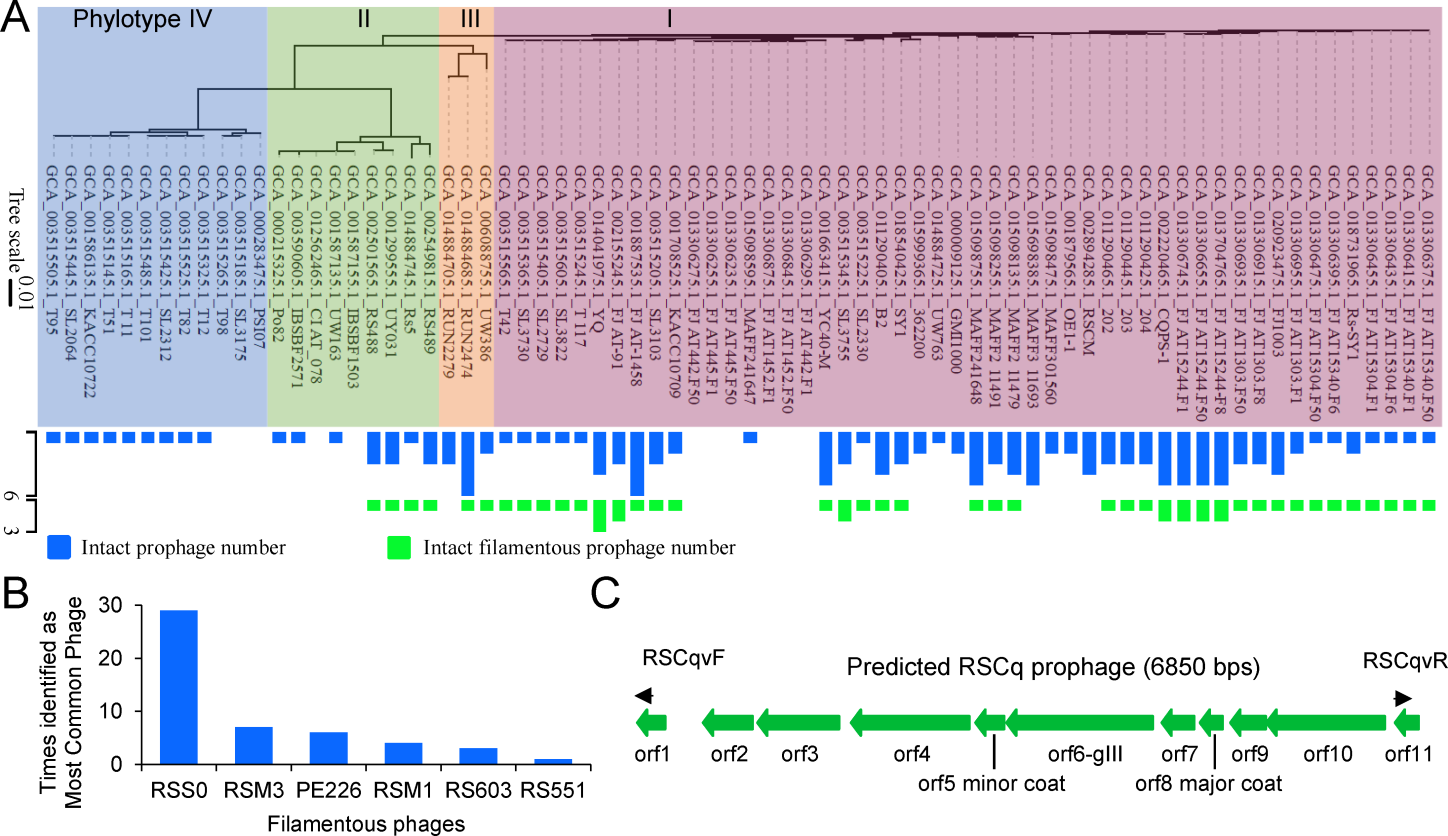
Prophage mining in the genomes of *R. solanacearum*. (**A**) Prophages were predicted *via* PHASTER. The genomic phylogenetic tree of *R. solanacearum* strains was constructed *via* GToTree based on the single-copy gene set. The number of predicted prophages and the phylogenetic tree were visualized *via* tvBOT. (**B**) Times of filamentous phages identified as “Most Common Phage” which is defined by the phage(s) with the highest number of proteins most similar to those in the identified prophage. (**C**) A filamentous prophage sequence was identified in the *R. solanacearum* phylotype I strain Cq05 isolated from grafted chieh-qua at Nanning, Guangxi Province, China.

Many filamentous phages can integrate into the host chromosome and replicate with the bacterial genome (11). In this study, 50 phylotype I, 9 phylotype II, 3 phylotype III, and 12 phylotype IV *R. solanacearum* strains with publicly available completed genomes were analyzed to evaluate the diversity of integrated prophages using the phage search tool PHASTER (15). As shown in Fig. 1A; Table S1, at least one intact prophage sequence was found in 63 of the 74 *R*. *solanacearum* strains, resulting in 152 intact prophage sequences in the total strains. Among these 152 sequences, 50 encode filamentous phages, based on the result of the “Most Common Phage,” which is an important term in PHASTER defined by the phage(s) with the highest number of proteins most similar to those in the identified prophage. Filamentous phage RSS0 (sequence accession: NC_019548) that infects *R. solanacearum* was most frequently identified as the “Most Common Phage” (29/50), followed by RSM3 (7/50) and PE226 (6/50) that infects *R. solanacearum* (Fig. 1B; Table S1). The 50 filamentous prophage sequences are distributed in 42 *R*. *solanacearum* strains (36 phylotype I strains, 4 phylotype II strains, and 2 phylotype III strains). These results suggest that filamentous prophages are distributed widely throughout *R. solanacearum* phylotypes I, II, and III strains, underscoring the feasibility of isolating filamentous phages that infect *R. solanacearum* through genome mining approaches.

*R. solanacearum* phylotype I (*R. pseudosolanacearum*) strain Cq05 isolated from grafted chieh-qua in Nanning, Guangxi Province, China, was genome sequenced to discover filamentous phage and the genome sequence was deposited at GenBank (BioProject ID PRJNA974909). As indicated in Table 1, five prophage regions, with three intact regions (region1, region2, and region5), were identified in the genome of Cq05. The “Most Common Phage” of region1 and region2 are RSY1 and RSA1, respectively. RSY1 and RSA1 belong to the *Myoviridae* family of phages that infect *R. solanacearum*. As shown in Fig. 1C, the 6.8 kb region5 sequence in contig JASKHZ010000087.1 was predicted to be an intact prophage. This prophage sequence encodes 11 open reading frames (ORFs), all of which are homologous to that of the reported *R. solanacearum* filamentous phage RSS1 (16) (sequence accession: NC_008575). *orf5* encodes a putative minor coat protein, while *orf8* encodes a putative major coat protein (pVIII) of filamentous phages. *orf6* encodes a putative pIII protein, which together with pVI, caps the terminal end of filamentous phages. These findings suggest that prophage region5 may encode a potential filamentous phage, which is named RSCq.

**TABLE 1:** Prophage regions identified in *R. solanacearum* Cq05

Region	Region length	Completeness	# Total proteins	Most Common Phage
1	40.5 Kb	Intact	41	Ralsto_RSY1_NC_025115(35)
2	38.3 Kb	Intact	37	Ralsto_RSA1_NC_009382(30)
3	8.6 Kb	Incomplete	8	Agroba_Atu_ph07_NC_042013(2)
4	5.1 Kb	Incomplete	8	Ralsto_RsoM1USA_NC_049432(2)
5	6.8 Kb	Intact	11	Ralsto_RSS1_NC_008575(11)

### Characterization of the filamentous phage RSCq

**Fig 2:**
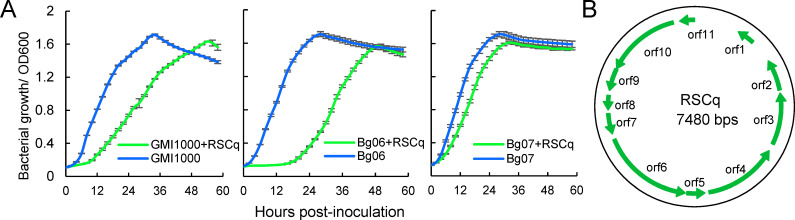
Characterization of the filamentous phage RSCq. (**A**) Effect of filamentous phage RSCq infection on the growth of *R. solanacearum* GMI1000, Bg06, and Bg07. The growth curves of *R. solanacearum* strains with or without RSCq infection were measured by monitoring bacterial growth (A600) *via* Bioscreen C Pro. The error bar is represented by the standard deviation of three technical repeats. The growth curves were assayed three times independently. (**B**) The replicative form genome of filamentous phage Cq05.

To isolate the filamentous phage RSCq, we took advantage of the cooperative relationship between filamentous phages and their bacterial hosts, in which filamentous phages are continuously secreted from the bacterial hosts. We initially detected the presence of RSCq in the culture supernatant of Cq05 *via* double agar overlay plaque assay using *R. solanacearum* GMI1000 as host bacteria. Small and turbid plaques were observed. Subsequently, two rounds of single plaque picking up and infection were performed to purify plaque. The isolated filamentous phage RSCq was cultured using GMI1000 as host bacteria. RSCq in GMI1000 was verified *via* polymerase chain reaction (PCR) using primers RSCqvF and RSCqvR (Table S3). In Fig. 2A, the filamentous phage RSCq was inoculated with tested host bacteria at a multiplicity of infection (MOI) of 10. *R. solanacearum* phylotype I Bg06 and Bg07 were isolated from bitter gourd in Guangxi Province, China. The presence of RSCq significantly delayed the growth of *R. solanacearum* GMI1000, Bg06, and Bg07, but might not lyse them. This finding is consistent with the biological features of filamentous phages.

Filamentous phages typically have a circular ssDNA genome within their virions, but a double-stranded replicative form (RF) of the genome exists in the host bacteria during the phage life cycle (11). The RF DNA of RSCq was then extracted *via* the plasmid DNA purification procedure. To verify the RF DNA of RSCq and compare the genome sequence of RSCq with the predicted genome, we amplified and sequenced the flanking sequence of the junction site using primers that bind to *orf1* and *orf11* (Fig. 1C). Unexpectedly, the amplification product is 925 bps in length, which is larger than the deduced 295 bps based on the PHASTER analysis, suggesting that the genome of RSCq is larger than initially predicted. The whole genome of RSCq was then sequenced *via* Sanger sequencing (10.6084 /m9.figshare.24473443). The genome was corrected to 7,480 bps (Fig. 2B) and was deposited in GenBank (Accession number: OR088903).

### A novel method for engineered phages construction

We assayed the effect of phage RSCq infection on the virulence of *R. solanacearum* through the stem injection of tomato and tobacco plants. No remarkable virulence difference was observed between phage-infected and uninfected *R. solanacearum* (Fig. 7; Fig. S4). When *R. solanacearum* infected tobacco *via* natural inoculation, RSCq infection delayed bacterial wilt symptoms but resulted in a similar final disease index of tobacco plants (Fig. 8). This result prompted us to construct engineered phages that are capable of delivering biocontrol factors.

**Fig 3:**
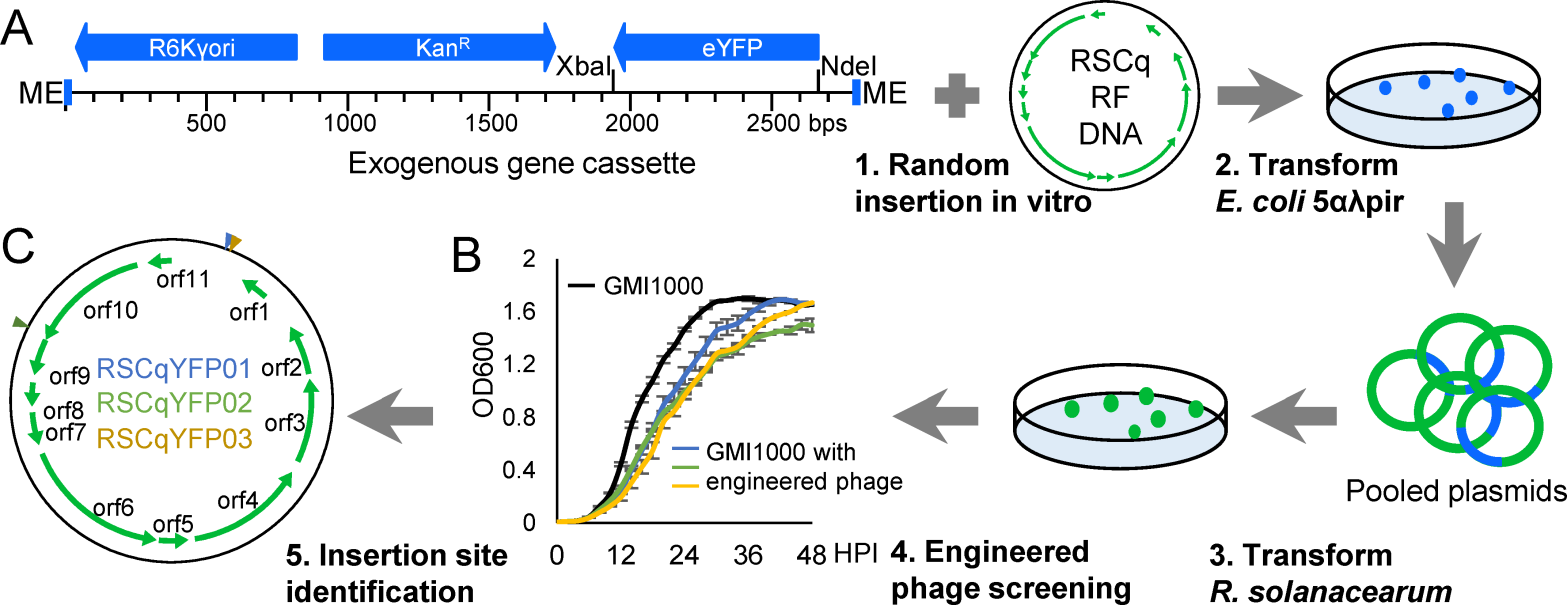
Construction of engineered phages based on RSCq. (**A**) Construction procedure of engineered phages based on RSCq. The exogenous gene cassette containing the R6Kγori sequence, kanamycin resistance gene (Kan^R^), *eYFP* gene, and the transposase recognition sequence (ME) was randomly inserted into RSCq replicative form DNA *in vitro* by Tn5 transposase, generating a plasmid library. The pooled plasmids library was then transformed into *R. solanacearum* GMI1000 to recover engineered phages. (**B**) Growth curve of *R. solanacearum* GMI1000 infected by engineered phages. The error bar is represented by the standard deviation of three technical repeats. The growth curves were assayed three times independently. (**C**) The exogenous gene cassette insertion site of engineered phage RSCqYFP01, RSCqYFP02, and RSCqYFP03.

The insert site of an exogenous target gene is crucial for phage genetic engineering, which relies on comprehensive studies of phage functional genomics. We proposed a novel method for non-model phage genetic engineering that involved propagating RSCq RF DNA as an independently replicating plasmid in *Escherichia coli* using Tn5 transposase. As depicted in Fig. 3A, the modified transposon of the EZ-Tn5 < R6Kγori/KAN-2 > Insertion Kit (Lucigen, Wisconsin, USA) was used as the exogenous gene scaffold. The modified transposon contained the *eYFP* gene controlled by the lac promoter, which was inserted between the transposase recognition sequence (ME) and the kanamycin resistance gene (KanR). The exogenous gene cassette was then randomly inserted into RSCq RF DNA *in vitro* by Tn5 transposase. The resulting transposon-inserted plasmid library was electrotransformed into *R. solanacearum* GMI1000, followed by engineered phage screening based on the growth inhibition effect. The supernatant of three transformants showed a growth inhibition effect on GMI1000, based on the growth curve (Fig. 3B). This finding suggests that the engineered phages were secreted from these transformants and named RSCqYFP01, RSCqYFP02, and RSCqYFP03.

The inserted site in the genome of the engineered phages was determined *via* Sanger sequencing using primers that bind on the transposon. Consequently, the insertion sites were identified for each engineered phage (Fig. 3C). In the case of RSCqYFP01, the exogenous genes were inserted into the noncoding regions downstream of *orf1*, particularly at positions 553–554 bp of the RSCq RF DNA. For RSCqYFP03, the exogenous genes were inserted at positions 561–562 bp. RSCqYFP02 had its exogenous genes inserted at positions 6,168–6,169 bp of the RSCq RF DNA, which is located at the 3′ end of *orf10*.

### Infectious feature of engineered phage RSCqYFP01

**Fig 4:**
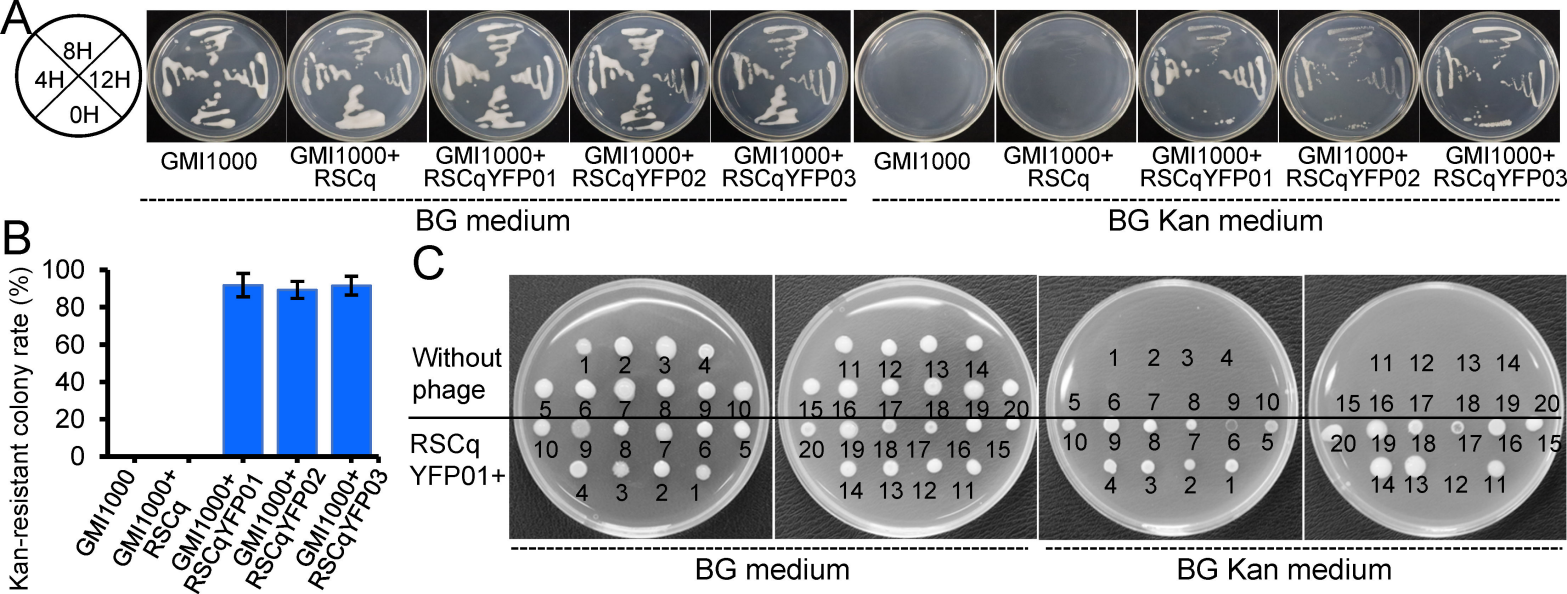
Infectious feature of engineered phage. (**A**) *R. solanacearum* streaked on BG agar medium with or without kanamycin at 0, 4, 8, and 12 h after the infection of the phage RSCq or the engineered phages RSCqYFP01, RSCqYFP02, or RSCqYFP03. (**B**) The kanamycin-resistant colony rate of *R. solanacearum* plating on BG agar medium with or without kanamycin 12 h post-infection of the phage RSCq or the engineered phages. The error bar is represented by the standard deviation of three technical repeats. This experiment was performed three times independently. (**C**) Host range of the engineered phage RSCqYFP01. The tested *R. solanacearum* strains (Table S2) were cultured on BG agar medium with or without kanamycin 12 h post-infection of the phage RSCq or the engineered phages.

Infectious capability was assayed to confirm the engineered filamentous phages. The engineered filamentous phages RSCqYFP01, RSCqYFP02, and RSCqYFP03 were inoculated with *R. solanacearum* GMI1000 in BG medium. The kanamycin resistance gene present in the engineered filamentous phages enabled the identification of infected *R. solanacearum* cells. As depicted in Fig. 4A, bacterial culture at various time points (0, 4, 8, and 12 h) after inoculation was streaked onto BG agar medium with or without kanamycin, and the result showed that *R. solanacearum* acquired kanamycin resistance when co-cultured with RSCqYFP01, RSCqYFP02, or RSCqYFP03. The infectious efficiency 12 h post-inoculation was quantified by the colony-forming unit on BG agar medium with or without kanamycin. As shown in Fig. 4B, more than 85% of *R. solanacearum* cells exhibited resistance to kanamycin after 12 h of engineered filamentous phage infection. These findings signify the efficient infectivity of the engineered filamentous phages in host bacteria. Prototype “Trojan Horse” viruses were constructed successfully. Among the engineered phages, RSCqYFP01 was selected for further research. Figure S1 provides the map of RSCqYFP01, while additional details regarding the sequence are shown in Supplementary Text 1.

In addition to *R. solanacearum* GMI1000, 19 *R*. *solanacearum* phylotype I strains isolated from diverse plant hosts in various locations within Guangxi, China, were tested to determine the host range of the engineered filamentous phage. Detailed information regarding the 19 strains is provided in Table S2. RSCqYFP01 and *R. solanacearum* strains were cocultured in BG medium for 12 h, followed by kanamycin resistance assay. As shown in Fig. 4C, RSCqYFP01 successfully infected 19 out of 20 of the tested strains, indicating that the engineered filamentous phage exhibits a broad host range on *R. solanacearum* phylotype I strains.

### Target gene can be delivered to host bacteria by engineered phage

**Fig 5:**
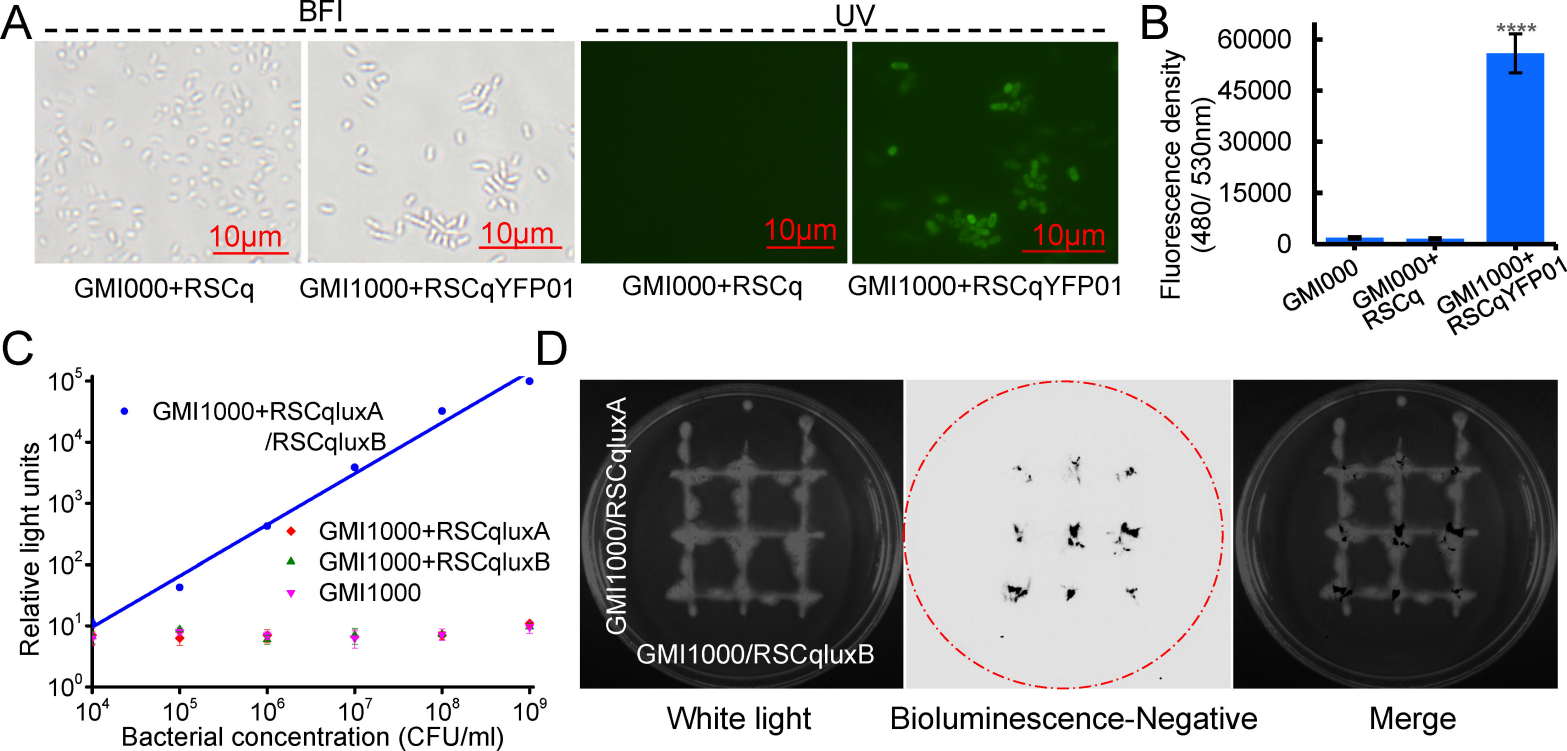
Target genes can be delivered into host bacteria by engineered phage. (**A**) Engineered filamentous phage RSCqYFP01 infected *R. solanacearum* GMI1000 under a fluorescence microscope. BFI, bright field images. UV, images under ultraviolet excitation. (**B**) The fluorescence density of the engineered filamentous phage RSCqYFP01 infected *R. solanacearum* GMI1000. The error bar is represented by the standard deviation of three technical repeats. This experiment was performed three times independently. (**C**) Relative light units of *R. solanacearum* GMI1000 co-infected by engineered filamentous phages RSCqluxA and RSCqluxB. RSCqluxA or RSCqluxB infected *R. solanacearum* GMI1000, and GMI1000 without phage infection were used as controls. The error bar is represented by the standard deviation of three technical repeats. This experiment was performed three times independently. (**D**) Luminescence imaging of the engineered filamentous phages RSCqluxA- and RSCqluxB- infected *R. solanacearum* (RSCqluxA/GMI1000 and RSCqluxB/GMI1000, respectively) on BG agar medium. Left panel, image of RSCqluxA/GMI1000 and RSCqluxB/GMI1000 under white light. Middle panel, the negative image of bioluminescence in the dark. Right panel, merged image.

As mentioned above, an *eYFP* gene was introduced into the transposon when constructing the engineered phages. The inoculation of RSCqYFP01 and *R. solanacearum* GMI1000 in BG medium for 48 h resulted in a yellow-green fluorescence signal in the infected bacteria under a fluorescence microscope (Fig. 5A). By contrast, no fluorescence signal was observed in *R. solanacearum* infected by the parent phage RSCq. Measurements of fluorescence density using a detection reader showed only background fluorescence in non-infected and RSCq-infected strains; meanwhile, high fluorescence density was detected in the RSCqYFP01-infected strain (Fig. 5B).

We replaced the *eYFP* gene with *luxA* and *luxB* genes, generating engineered phage RSCqluxA and RSCqluxB, respectively, to confirm the delivery and expression of the exogenous gene further. The LuxAB heterodimeric enzyme, but not LuxA or LuxB alone, catalyzes the bioluminescence reactions emitting blue-green light (17). *R. solanacearum* GMI1000 was co-infected by engineered phages RSCqluxA and RSCqluxB in BG liquid medium for 24 h. The infected bacteria were subject to gradient dilution, and bioluminescence was measured after adding the luminescent substrate aldehyde. As shown in Fig. 5C, an intense bioluminescent signal was detected in RSCqluxA/RSCqluxB co-infected GMI1000, but not in wild-type GMI1000, RSCqluxA-, or RSCqluxB-infected GMI1000. The relative light units (RLUs) of RSCqluxA/RSCqluxB co-infected GMI1000 were correlated linearly with bacterial concentration. The infected bacteria were also diluted and plated on BG agar medium. The resulting colonies were imaged under a luminescence imaging system after spraying the luminescent substrate aldehyde. The luminescence of colonies indicated that RSCqluxA and RSCqluxB can co-infect a single *R. solanacearum* cell (Fig. S2).

RSCqluxA- and RSCqluxB- infected *R. solanacearum*, namely RSCqluxA/GMI1000 and RSCqluxB/GMI1000, respectively, were streaked on BG agar medium by crossing each other. Luminesce can be detected at the intersection of RSCqluxA/GMI1000 and RSCqluxB/GMI1000 after spraying of the luminescent substrate aldehyde (Fig. 5D), suggesting engineered phages can be continuously secreted from the infected *R. solanacearum* cells and infect other surrounding host cells. This finding demonstrates that exogenous genes integrated with the engineered phage genome can be delivered and expressed efficiently in the host bacterial cells along with phage infection. The soldiers, represented by the exogenous genes delivered by the “Trojan Horse,” effectively function within the cells of *R. solanacearum*.

### Engineered phage delivering CRISPR-AsCas12f1 targets *hrpB* of *R. solanacearum*

The hypersensitive response and pathogenicity (*hrp*) genes encoded type III secretion system, with its delivered type III effectors (T3Es), is one of the major virulence determinants of *R. solanacearum* (18). By disrupting the *hrpB* gene, a key regulator of *hrp* genes, namely, a mutant strain of *R. solanacearum* is avirulent and can function as a biocontrol agent against bacterial wilt caused by this pathogen (19, 20). We plan to deliver the CRISPR-Cas system that targets *hrpB* into *R. solanacearum* in nature *via* the engineered phage to disarm the key weapon of *R. solanacearum* and make the pathogen an avirulent biocontrol agent.

**Fig 6:**
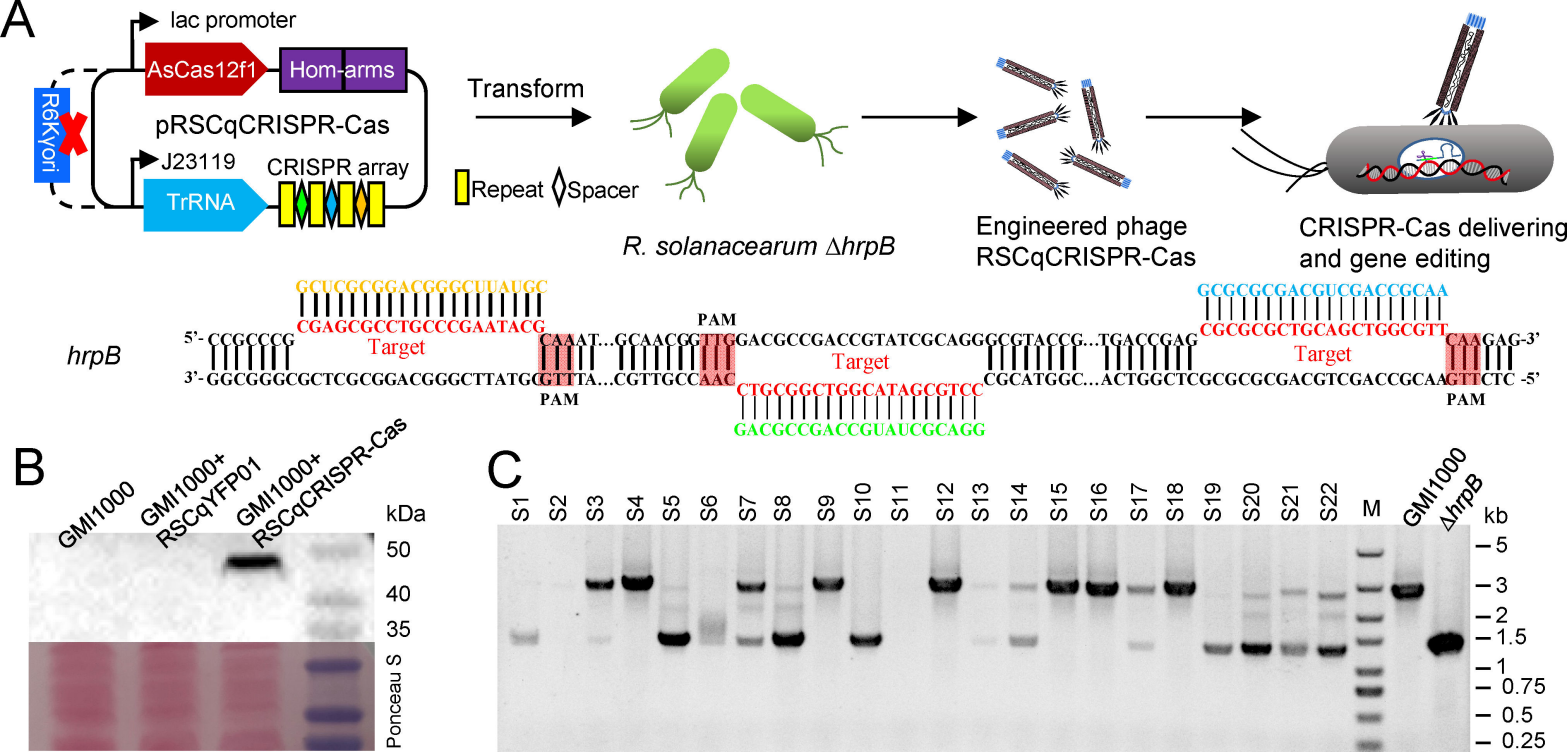
Engineered phage delivering CRISPR-AsCas12f targets *hrpB* of *R. solanacearum*. (**A**) Schematic of sgRNA scaffold design and engineered phage RSCqCRISPR-Cas construction. AsCas12f1, sgRNA, and two 400 bp homologous arms (Hom-arms) were cloned to the phasmid vector pRSCqYFP01. The R6kγori sequence of the resulting plasmid was removed *via* Gibson assembly to reduce the size of cargo DNA. The reaction sample of Gibson assembly was transformed into the *R. solanacearum* Δ*hrpB*, generating engineered phage RSCqCRISPR-Cas. Sequences targeted by CRISPR and protospacer adjacent motif (PAM) sequences are indicated. (**B**) AsCas12f1 (49.5 kDa expected) in the total protein of *R. solanacearum* GMI1000 infected with engineered phage RSCqCRISPR-Cas was detected *via* Western blot using a monoclonal antibody against the 6*His tag. GMI1000 and GMI1000 infected with RSCqYFP01 were set as control groups. (**C**) Verification of *hrpB* deletion mediated by engineered phage RSCqCRISPR-Cas *via* PCR using primers that bind the flanking sequence of the homologous arms. *R. solanacearum* GMI1000 was infected with RSCqCRISPR-Cas for 48 h in MP medium, plated on BG agar medium, and cultured at 37°C. *R. solanacearum* wild-type strain GMI1000 and mutant Δ*hrpB* were used as controls.

CRISPR-Cas9 system was cloned to the phasmid vector. However, no infective engineered phage was obtained, due to the limited cargo size of the engineered phage. A recently reported miniature class 2 type V-F CRISPR-Cas from *Acidibacillus sulfuroxidans* (CRISPR-AsCas12f1) (21, 22) was then selected for the in-nature gene editing. As shown in Fig. 6A, the AsCas12f1 gene was placed under the control of a lac promoter, and a 6*his tag was added. The single-guide RNA (sgRNA) was designed with three 20 bp spacers targeting 48–67 bp, 642–661 bp, and 1,210–1,229 bp of *hrpB* ORF. Two 400 bp homologous arms were designed to edit the *hrpB* targeted region because non-homologous DNA end joining (NHEJ) is lacking in *R. solanacearum*. AsCas12f1, sgRNA, and homologous arms were cloned to the phasmid vector pRSCqYFP01. However, the cargo was too large to produce an infective phage. The R6kγori sequence (780 bp) was removed using the Gibson assembly method for the assembly of three linear DNA fragments amplified by the primers listed in Table S3. The Gibson assembly reaction sample was directly transformed into the *R. solanacearum* mutant Δ*hrpB* that lacks target sequences of sgRNA to generate an infective engineered phage because the resulting DNA without R6kγori cannot replicate in *E. coli*. The resulting infective phage, which now contains the CRISPR-AsCas12f system, was named RSCqCRISPR-Cas (Fig. S3). An engineered phage without sgRNA and homologous arms, namely, RSCqCas, was also constructed for control.

*R. solanacearum* GMI1000 was infected with the engineered phage RSCqCRISPR-Cas for 24 h, and Western blot analysis using a monoclonal antibody against the 6*His tag was subsequently performed. As shown in Fig. 6B, AsCas12f1, which is expected to be 49.5 kDa, can be detected in the total protein of RSCqCRISPR-Cas infected strain but not in GMI1000 or RSCqYFP01infected stain, indicating the successful delivery and expression of the AsCas12f1 gene in *R. solanacearum cells*.

To assess the gene-editing effect of the CRISPR-AsCas12f1 system, *R. solanacearum* GMI1000 was infected with the engineered phage RSCqCRISPR-Cas for 48 h in BG and MP media. The resulting culture was diluted and plated on BG agar medium and incubated at 28°C or 37°C. The gene editing effect of CRISPR-AsCas12f1 was assessed *via* PCR using primers that bind the flanking sequences of the homologous arms. However, no gene editing was detected for RSCqCRISPR-Cas infection in the BG medium. When *R. solanacearum* was infected in MP medium and cultured at 28°C after spread plating, most randomly selected colonies showed bands for wild type and *hrpB* deletion. Wild type, *hrpB* deleted, hetero-type (showing bands for wild type and *hrpB* deletion), and potential off-target colonies without any bands were detected when cultured at 37°C (Fig. 6C). These findings indicate that the CRISPR-AsCas12f1 system successfully edited the target gene, *hrpB*, although the efficiency and accuracy of gene editing in *R. solanacearum* may not yet be sufficient for gene functional studies.

### Engineered phage RSCqCRISPR-Cas attenuates the virulence of *R. solanacearum*

**Fig 7:**
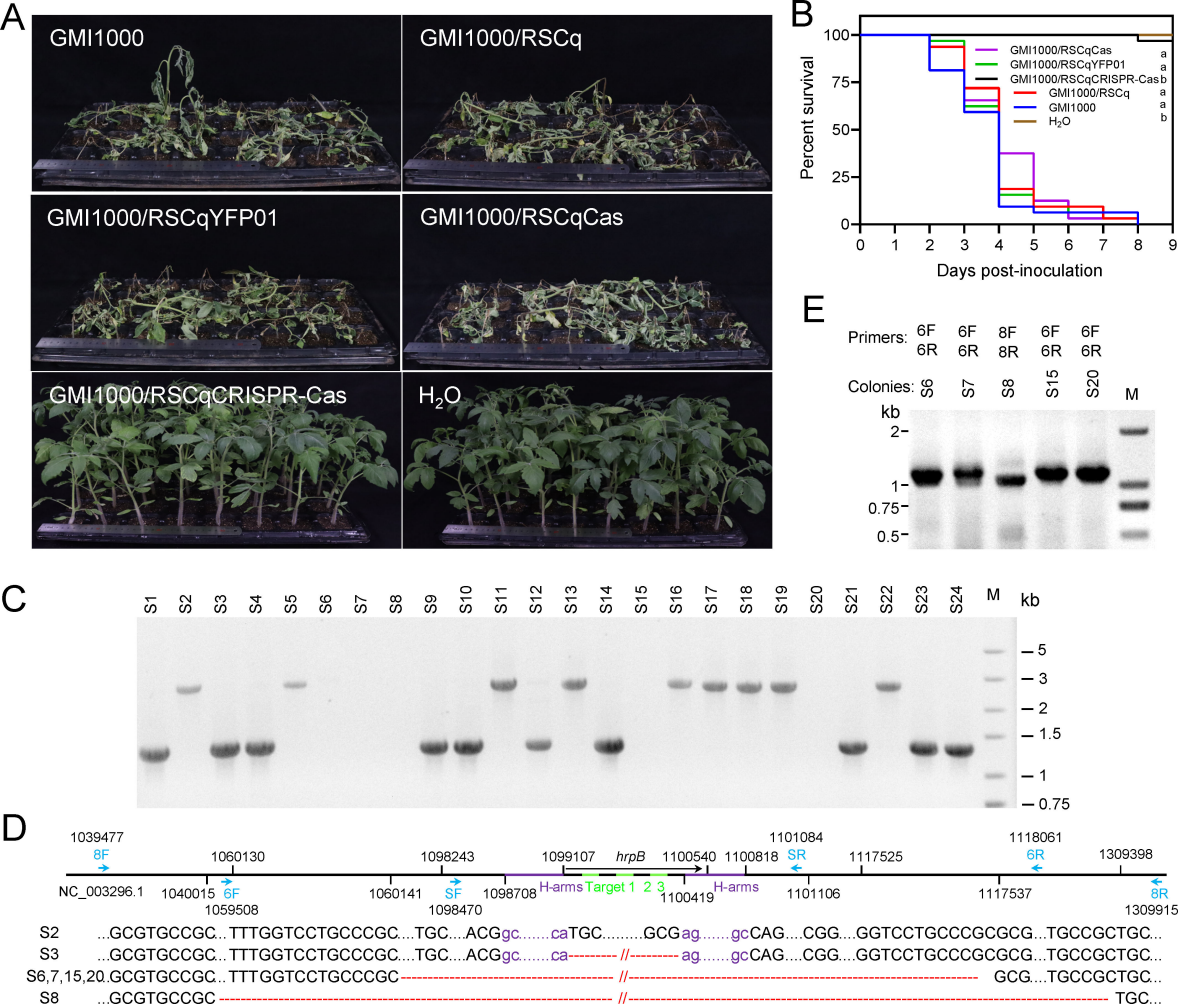
The engineered phage RSCqCRISPR-Cas attenuates the virulence of *R. solanacearum*. (**A**) Bacterial wilt symptoms of tomato plants 9 days after inoculation with *R. solanacearum* GMI1000, GMI1000 infected with phage RSCq or engineered phages. Virulence was assayed three times independently, and one representative result was shown. (**B**) Survival curve of infected tomato plants. Kaplan-Meier survival analysis with the Gehan-Breslow-Wilcoxon method was used to compare pathogenicity between the mutant and wild-type strains. Different letters represent significant differences at *P* < 0.05 probability level. (**C**) PCR verification of *hrpB* deletion mediated by the engineered phage RSCqCRISPR-Cas of colonies recovered from infected tomato plants. (**D**) Genome resequencing deduced gene deletion in plant-recovered colonies S2, S3, S6, S7, S8, S15, and S20. The red dashes correspond to deleted nucleotides in the corresponding mutants, while the dots represent conserved nucleotides. (**E**) PCR verification of gene deletion in plant-recovered colonies S6, S7, S8, S15, and S20. The primers used are marked in Fig. 7D.

*R. solanacearum* GMI1000 was infected with the engineered phage RSCqCRISPR-Cas for 12 h in BG medium, and no gene editing was detected by PCR verification after spread plating. The engineered phage-infected *R. solanacearum* was inoculated to susceptible tomato plants *via* stem injection to evaluate the effect on the virulence of *R. solanacearum*. As shown in Fig. 7A and B, nearly all the tomato plants inoculated with GMI1000, GMI1000/RSCq (RSCq-infected GMI1000), GMI1000/RSCqYFP01, and GMI1000/RSCqCas exhibited wilt symptoms 9 days post-inoculation. However, 96.9% of the tomato plants survived GMI1000/RSCqCRISPR-Cas, suggesting that RSCqCRISPR-Cas significantly attenuated the virulence of GMI1000.

*R. solanacearum* was then recovered from the stem of GMI1000/RSCqCRISPR-Cas-infected tomato plants *via* spread plating. PCR verification was performed on randomly selected colonies to further assay the gene editing effect of CRISPR-Cas12f. As shown in Fig. 7D, hetero-type colonies were not detected, unlike in the medium. Among the 24 randomly selected colonies, 10 showed bands indicating *hrpB* deletion, nine were wild-type strains, and no PCR product was obtained for the remaining five colonies. Whole-genome sequencing was performed on the five colonies that did not yield PCR products, along with a wild-type colony and an *hrpB*-deleted colony. The *hrpB* locus with its flanking sequence was aligned to the assembled genome of the sequenced colonies using BLASTn. As shown in Fig. 7D, genome resequencing confirmed that colonies S2 and S3 were wild type and *hrpB* mutant, respectively. Moreover, genome re-sequencing found that 57.4 kb of DNA flanking *hrpB* was deleted in colonies S6, S7, S15, and S20. Meanwhile, 269.4 kb of DNA flanking *hrpB* was deleted in colony S8. These deletions were further validated *via* PCR using primers that bind to the flanking sequences of the deduced deletion regions (Fig. 7E). All these findings indicate that CRISPR-AsCas12f-mediated gene editing persisted during plant infection of *R. solanacearum* and ultimately weakened its virulence on the host plant.

Tobacco (*Nicotiana tabacum*) Yunyan87, a major cultivar planted in China, was selected as the test host plant to confirm the effect of RSCqCRISPR-Cas on *R. solanacearum* virulence. The virulence of RSCqCRISPR-Cas-infected *R. solanacearum* phylotype I Tb04, which was isolated from tobacco in Baise, Guangxi, China (23), was determined *via* stem injection. Similarly, the virulence of *R. solanacearum* Tb04 on tobacco Yunyan87 was significantly attenuated by the infection of RSCqCRISPR-Cas (Fig. S4).

### Plant bacterial wilt can be efficiently controlled by the engineered phage RSCqCRISPR-Cas

**Fig 8:**
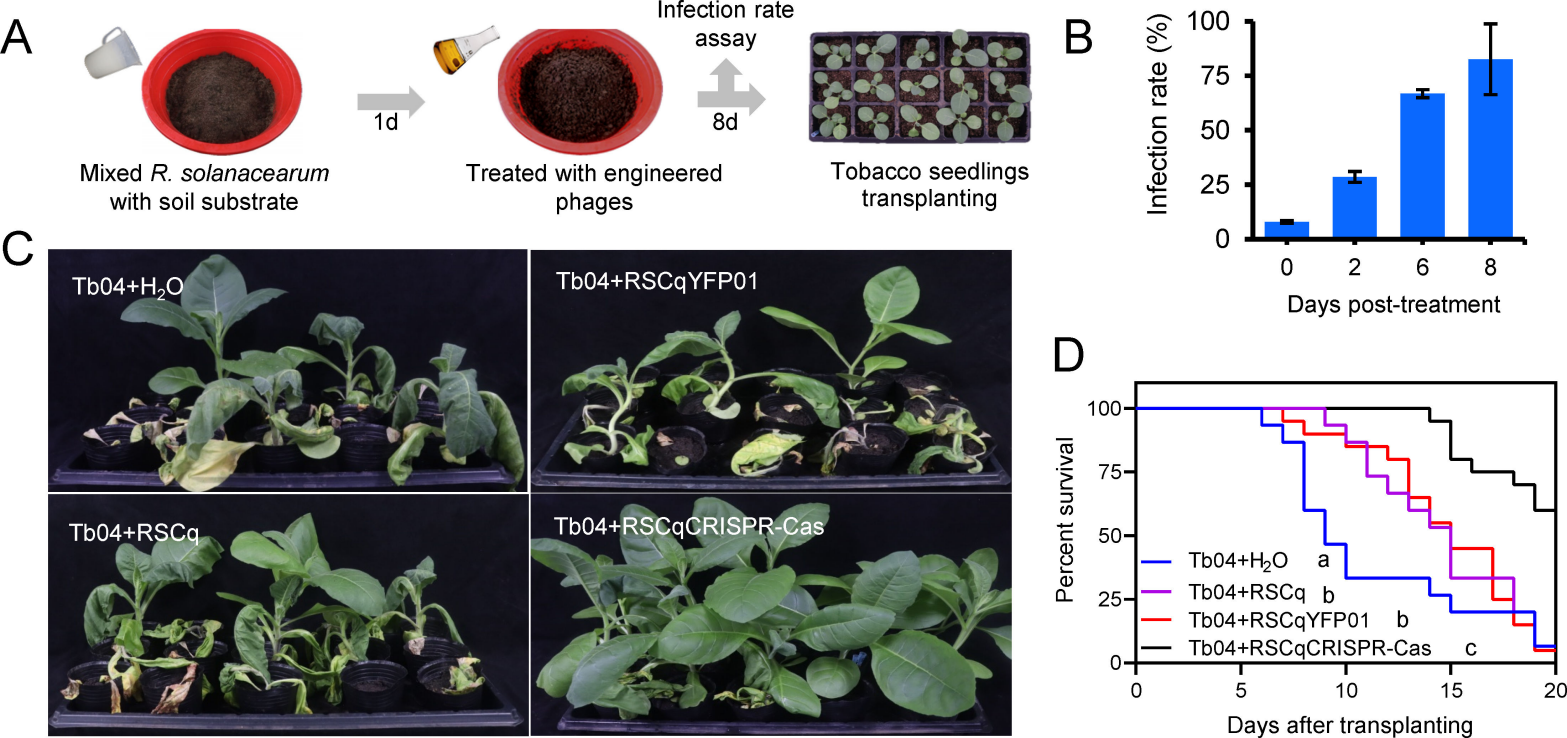
Plant bacterial wilt biocontrol effect of the engineered phage RSCqCRISPR-Cas. (**A**) Schematic of infection rate assay in soil and biocontrol assay of engineered phage. (**B**) The infection rate of RSCqYFP01 on *R. solanacearum* in soil substrate. The error bar is represented by the standard deviation of three technical repeats. This experiment was performed three times independently. (**C**) Bacterial wilt symptoms of tobacco 20 days after transplants to *R. solanacearum* Tb04 contaminated soils with or without phage treatment. The biocontrol experiment was assayed three times independently, and one representative result was shown. (**D**) Survival curve of tobacco planted in soils with or without phage treatment. Kaplan-Meier survival analysis with the Gehan-Breslow-Wilcoxon method was used to compare bacterial wilt of tobacco planted in different soils. Different letters represent significant differences at *P* < 0.05 probability level.

The infection efficiency of the engineered filamentous phage in soil was also assayed to evaluate its potential for biocontrol applications. As shown in Fig. 8A, *R*. *solanacearum* GMI1000 was introduced into a soil substrate at a final concentration of 10^8^ colony-forming units (CFU) per gram of substrate, followed by RSCqYFP01 treatment after 1 day of *R. solanacearum* watering. Based on the ratio of kanamycin-resistant colonies, 82.6% of *R. solanacearum* cells in the soil were infected after 8 days of RSCqYFP01 treatment (Fig. 8B). The “Trojan Horse” can be efficiently implanted into *R. solanacearum* in a soil environment.

Tobacco Yunyan87 was then used as the test host plant to explore the biocontrol effect of engineered phages in plant bacterial wilt. The target region of CRISPR-AsCas12f was conserved in the genome of *R. solanacearum* Tb04 (BioProject ID PRJNA616449), which was used as the tested pathogen. As shown in Fig. 8A, Tb04 was watered to soil substrate at a final concentration of 10^8^ CFU/g substrate, followed by engineered phage infection 1 day after *R. solanacearum* watering. The soil substrate was kept wet for 8 days to fully infect *R. solanacearum*. Then 3-week-old tobacco seedlings were transplanted to Tb04 contaminated soil with or without phage treatment. Although phage RSCq and engineered phage RSCqYFP01 treatment delayed bacterial wilt symptoms, the final disease index of tobacco planted in RSCq- or RSCqYFP01-treated soil was similar to that of tobacco planted in soil without treatment (Fig. 8C and D). However, the survival percentage of tobacco planted in soil with engineered phage RSCqCRISPR-Cas treatment was significantly higher than that of tobacco planted in other soil. RSCqCRISPR-Cas treatment efficiently controlled the bacterial wilt of tobacco with a biocontrol efficiency of 59.2%. That is, the “Trojan Horse” viruses that deliver CRISPR-AsCas12f help protect plants from the pathogen *R. solanacearum*.

## DISCUSSION

### More filamentous phages can be efficiently isolated via genome mining

Filamentous phages, regarded as masters of a microbial sharing economy, play crucial roles in promoting bacterial virulence, shaping bacterial communities, and promoting biotechnology developments (11). More filamentous phages should be discovered to make the study of filamentous phages flourish. A study used a machine learning approach to mine microbial genomes and metagenomes for inoviruses. A total of 10,295 inovirus-like sequences were found, from which 5,964 distinct species appear to have been identified (24). This finding alone represents a 100-fold expansion of the previously described diversity (57 genomes) within the *Inoviridae* family (24, 25). These results indicate a vast pool of unexplored filamentous phages that await functional analysis. Our study identified intact filamentous prophage sequences in 42 of the 62 investigated *R. solanacearum* phylotypes I, II, and III genomes, suggesting that filamentous prophages distributed widely throughout *R. solanacearum* phylotypes I, II, and III strains. A filamentous phage was subsequently successfully isolated *via* genome mining. We believe that more filamentous phages that infect *R. solanacearum* or other bacteria can be efficiently isolated *via* genome mining, because of the low cost of bacterial genome sequencing.

### Proposed filamentous phage engineering method should be useful

Phage genetic engineering enables deliberate modifications of natural phage isolates to enhance their suitability for various applications (26). For example, M13-based engineered phage delivers the CRISPR-Cas system that targets the carbapenem-resistant gene of enterohemorrhagic *E. coli* and significantly improves survival in a *Galleria mellonella* infection model (27). Staphylococcal phage ΦNM1-based delivers the CRISPR-Cas system that targets antibiotic-resistant genes and functions *in vivo* to kill *S. aureus* in a mouse skin colonization model (28). The application of phage-delivered CRISPR-Cas system in agriculture has not yet been reported, and engineering methods for non-model phage are important for this application. Moreover, the aforementioned M13-based and ΦNM1-based engineered phages mentioned cannot be secreted from infected bacteria, and engineered phage particles should be prepackaged with the m13cp helper plasmid or in ΦNM1 capsids. However, engineered phage replication from infected bacteria in nature is important for application in agriculture.

Many sophisticated phage genetic engineering methods have been developed (29). One such method is phage recombineering with electroporated DNA. Other approaches to phage modification include the assembly of an engineered phage genome from DNA fragments *in vitro*, followed by recovery of the engineered phage *via* the transformation of a suitable bacterial host (29). However, the insert site of an exogenous target gene is crucial for *in vitro* genome assembly. Previous methods relied on comprehensive studies of the functional genomics of specific phages, making them less applicable to non-model phages. Our filamentous phage engineering method proposed in this study followed the aforementioned strategy. We used Tn5 transposase to insert the modified transposon randomly into RSCq RF DNA *in vitro*, followed by engineered phage recovery. This approach is straightforward to implement and can be employed to genetically engineer other filamentous phages without extensive functional genomic studies.

### Superinfection of *R. solanacearum* is allowed for RSCq

Temperate phages typically encode superinfection exclusion mechanisms to prevent host lysis by virions of the same or similar species. For example, a filamentous phage protein PfsE inhibits type IV pili to prevent superinfection of *Pseudomonas aeruginosa* (30). In the current study, we show that *R. solanacearum* strain Cq05, which was infected by phage RSCq, can subsequently be infected by the engineered phage RSCqYFP01 and acquire kanamycin resistance. In addition, we observed that the engineered phages RSCqluxA and RSCqluxB can infect a single *R. solanacearum* cell and induce luminescence in the presence of a luminescent substrate. These results strongly suggest that superinfection of the tested *R. solanacearum* strains is permissible for the phage RSCq. The absence of phage protein PfsE permits superinfection by other type IV pili-dependent phages in *P. aeruginosa* (30), whereas the superinfection mechanism in *R. solanacearum* is yet to be elucidated. Moreover, the superinfection makes it possible to deliver larger exogenous DNA overcoming the limitation of cargo into *R. solanacearum* separately by multiple engineered phages.

### More biocontrol factors may be delivered by engineered filamentous phages

The CRISPR-AsCas12f1 gene editing system was used as a therapeutic payload of engineered phages in this study. CRISPR-AsCas12f1 is a novel mini gene editing system that has been recently reported to overcome the limited delivery size of viral-based vectors (22, 31). However, *R. solanacearum* gene editing mediated by engineered phage-delivered CRISPR-AsCas12f1 was modestly detected when culturing in minimal medium MP but was not detected when culturing in rich medium BG. This phenomenon may be related to the slower growth of *R. solanacearum* in the minimal medium. Studies found that, overall, the bacterial doubling times are positively correlated with the activities of CRISPR-Cas systems (32, 33). The gene editing efficiency and accuracy of CRISPR-AsCas12f1 in *R. solanacearum* should be optimized further. Recently, an engineered hypercompact CRISPR-Cas12f system based on AsCas12f has been demonstrated to be 11.3-fold more potent than the parent protein (34). Engineered AsCas12f and other miniature gene editing systems, such as Cas12n (35), can be potential plant bacterial disease biocontrol factors delivered by engineered filamentous phage. Further testing is necessary to evaluate their biocontrol effects.

Using resistant cultivars is considered the most effective strategy for controlling bacterial wilt and other plant diseases (36, 37). However, the *R. solanacearum* species complex is highly heterogeneous in nature, posing challenges to developing crop disease resistance. For example, in tomatoes, the polygenic resistance to bacterial wilt in the resistant cultivar Hawaii7996 was suggested to be strain-specific (38). Type III secreted effectors of *R. solanacearum* play key roles in crop disease resistance, and the diversity of type III secreted effectors in *R. solanacearum* species complex significantly impeded disease resistance breeding efforts (39, 40). The gene delivery strategy employed in this study can be applied to regulate the effectome of *R. solanacearum* species complex in nature to facilitate disease resistance breeding. For example, certain avirulence genes recognized by the resistance gene of crop cultivars can be delivered to *R. solanacearum* by an engineered filamentous phage, thereby converting the virulent *R. solanacearum* strain to an avirulent strain. However, many effectors are avirulent and virulent dual-functional. The potential risks of delivered avirulence genes should be fully evaluated.

### Biocontrol effect in a field with a long timescale should be further studied

We demonstrated that engineered phage that delivers CRISPR-AsCas12f controlled bacterial wilt of tomato and tobacco plants efficiently in a greenhouse. However, the field environment is more complex than a controlled greenhouse setting. Therefore, the biocontrol effect of the engineered phage on bacterial wilt in field conditions remains uncertain and requires further investigation. Bacterial resistance to phage infection is a significant obstacle in the widespread application of lytic phage reagents. By contrast, filamentous phages establish cooperative relationships with their bacterial hosts and exert minimal burden on them. However, interaction studies between filamentous phages and their host bacteria remain limited, and the occurrence pattern of bacterial resistance to filamentous phages is still unclear. Studying the long-term biocontrol effect of engineered phages is also an important area for future research.

Notably, the World Economic Forum has awarded designer phages as one of the Top 10 Emerging Technologies of 2023. Engineered phage therapeutics in animal bacterial infections have been widely studied and have shown their feasibility (26, 27, 41, 42). However, engineered phage biocontrol in plant diseases has been largely overlooked. Our study demonstrated the efficient control of plant bacterial wilt using engineered phages. The proposed phage engineering method is universal and suitable for non-model phages, and we believe the biocontrol strategy in this study can also be applied to other plant bacterial diseases. Further research in this area holds considerable promise.

## MATERIALS AND METHODS

### Bacterial strains and growth conditions

Phylotype I *R. solanacearum* GMI1000 (43) was mainly used as the model strain. *R. solanacearum* Cq05 (BioProject ID PRJNA974909) and other *R. solanacearum* strains were isolated from Guanxi, China, and are detailed in Table S2. *R. solanacearum* strains were cultured at 28°C in BG medium (10 g/L bactopeptone, 1 g/L casamino acids, 1 g/L yeast extract, and 5 g/L glucose) or on BG agar medium (44), unless otherwise specified. *Escherichia coli* DH5αλpir was used as the host strain for the replication of phagemid vectors and cultured at 37°C in LB medium or on LB agar medium. Kanamycin was supplemented when needed at a final concentration of 25 µg/mL.

### Phage mining and characteristic assay

Prophage sequences in *R. solanacearum* strains with complete genomes were downloaded from the Pre-Calculated Genome in PHASTER (15). The genome of *R. solanacearum* Cq05 (BioProject ID PRJNA974909) was submitted to PHASTER to predict prophage sequences. Filamentous prophages were determined by “Most Common Phage,” which is an important term in PHASTER defined by the phage(s) with the highest number of proteins most similar to those in the region. The genomic phylogenetic tree of *R. solanacearum* strains with a complete genome was constructed *via* GToTree based on the single-copy gene set (45) and visualized *via* tvBOT (46).

The filamentous phage RSCq was isolated using a double agar overlay plaque assay. Briefly, bacterial supernatant from the 12 h cultured *R. solanacearum* Cq05 was collected by centrifugation and filtered through a 0.22-µm membrane. The filtered supernatant was then serially diluted to achieve an appropriate plaque count on plates. The bacterial culture of host strain GMI1000 and the diluted supernatant which contains phage RSCq were mixed in 3 mL soft agar BG medium at 50°C. The mixture was quickly poured onto a hard agar BG medium surface and cultured at 28°C. The single plaque was picked into sterile SM buffer (100 mmol/L NaCl; 8 mmol/L MgSO_4_; 50 mmol/L Tris.HCl; 0.01% Gelatin) for a new round of double agar overlay plaque assay.

The number of plaque forming units (PFU) of filamentous phage RSCq in the supernatant of RSCq-infected GMI1000 was determined by double agar overlay plaque assay. The supernatant containing RSCq was added with a multiplicity of infection (MOI) of 10 and co-inoculated with the tested *R. solanacearum* strains. The growth curves of *R. solanacearum* strains with or without RSCq were measured every 2 hours monitoring bacterial growth (OD600) *via* Bioscreen C Pro (Oy Growth Curves Ab Ltd., Turku, Finland) with three technical replicates and three biological repeats.

The replicative form (RF) DNA of the phage RSCq in the RSCq-infected GMI1000 was extracted by alkaline lysis method, followed by phenol extraction. The flanking DNA of the RSCq junction site was amplified using primers RSCqvF and RSCqvR (Table S3) to confirm the isolated filamentous phage. The genome sequence of phage RSCq was determined by Sanger sequencing.

### Construction of engineered phages

The *eYFP* gene controlled by the lac promoter was inserted between the transposase recognition sequence (ME) and the kanamycin resistance gene (KanR) of the transposon of the EZ-Tn5 < R6Kγori/KAN-2 > Insertion Kit (Lucigen, Wisconsin, USA) *via* overlap extension PCR using primers list in Table S3. The modified transposon was then inserted into RSCq RF DNA randomly *in vitro* by Tn5 transposase according to the manufacturer’s instructions. The resulting DNAs were transformed to *E. coli* DH5αλpir, generating a transposon-inserted plasmids library. The transposon-inserted plasmid library was electrotransformed into *R. solanacearum* GMI1000, and the engineered phages were screened based on the growth inhibition effect of RSCq on *R. solanacearum* GMI1000.

The *luxA* and *luxB* genes were designed to be under the control of the promoter of the kanamycin resistance gene from the plasmid pK18mobsacB (47). The *luxA* and *luxB* gene cassettes were cloned to phagemid vector pRSCqYFP01 at the NdeI and XbaI sites using the primers listed in Table S3. The recombined vectors were electrotransformed into *R. solanacearum* GMI1000, and the engineered filamentous phages RSCqluxA and RSCqluxB were isolated from the supernatant of transformants.

AsCas12f1 and sgRNA scaffold were synthesized at GeneCreate Biological Engineering Co., Ltd, Wuhan, China. Homologous arms were amplified from *R. solanacearum* GMI1000. sgRNA scaffold and homologous arms were fused by overlapping PCR using primers listed in Table S3. The fused DNA fragment and AsCas12f1 gene were cloned to XbaI/NdeI- digested pRSCqYFP01 *via* Gibson assembly. The resulting plasmid was PCR amplified using primers listed in Table S3, generating three DNA fragments to remove R6Kγori. These DNA fragments were assembled *via* Gibson assembly, followed by transformation to *R. solanacearum* Δ*hrpB*. The engineered phage RSCqCRISPR-Cas was isolated from the supernatant of transformants. The map and the sequence of the engineered phage RSCqCRISPR-Cas RF DNA are available in Fig. S2 and Supplementary Text 2, respectively.

### Luminescence assay of *R. solanacearum* infected by engineered phages

The engineered phages RSCqluxA and RSCqluxB were obtained by centrifugation and filtered through a 0.22-µm membrane from the culture supernatant of infected *R. solanacearum* GMI1000. Phage-free *R. solanacearum* GMI1000 was co-infected by the engineered phages RSCqluxA and RSCqluxB in BG liquid medium for 24 h. The infected bacteria were then subjected to gradient dilution and quantification by colony counting. 1% aldehyde in ethyl alcohol was added to the diluted bacterial culture by 2%, and bioluminescence was detected using a Synergy II multiplate detection reader immediately. This process was performed with three biological repeats, and the average value ± standard deviation was presented.

GMI1000 infected by RSCqluxA-, RSCqluxB-, or co-infected by RSCqluxA/RSCqluxB were spread plated on BG agar medium after gradient dilution and cultured at 28°C. Subsequently, 1% aldehyde in ethyl alcohol was evenly sprayed on the surface of the culture. The resulting plate was immediately imaged under a luminescence imaging system (48). Bioluminescence images were inverted (i.e., photographic negatives were generated) and merged with the images taken under white light. This experiment was repeated three times, and representative images were presented. *R. solanacearum* GMI1000 was infected by the engineered phages RSCqluxA and RSCqluxB, generating RSCqluxA/GMI1000 and RSCqluxB/GMI1000, respectively. RSCqluxA/GMI1000 and RSCqluxB/GMI1000 were streaked on BG agar medium crossing each other and cultured at 28°C. The resulting cultures were imaged under a luminescence imaging system as described.

### Western blot analysis

*R. solanacearum* GMI1000 was infected by the engineered phage RSCqCRISPR-Cas for 24 h. The total protein of the resulting culture was extracted by boiling, followed by sodium dodecyl sulfate (SDS)-polyacrylamide gel electrophoresis (PAGE). The target protein was detected by Western blot after transferring the protein from the gel to the membrane. 6*His-tag monoclonal antibody was commercially acquired from Proteintech, Wuhan, China, and was used as a primary antibody for Western blot at a dilution of 1:10,000. HRP-conjugated Rabbit anti-mouse IgG was commercially acquired from Sangon, Shanghai, China, and was used as a secondary antibody at a dilution of 1:10,000. The resulting membrane was treated with an ECL luminescence reagent (Sangon, Shanghai, China) and imaged under a luminescence imaging system.

### RSCqCRISPR-Cas mediated gene editing assay

Engineered phage RSCqCRISPR-Cas was obtained by centrifugation and filtered through a 0.22-µm membrane from the culture supernatant of infected *R. solanacearum* Δ*hrpB. R. solanacearum* wild-type strain GMI1000 was infected by the engineered phages RSCqCRISPR-Cas in BG or MP (MP medium for 1L: 1.25 × 10^−4^ g FeSO_4_·7H_2_O, 0.5 g (NH_4_)_2_SO_4_, 0.05 g MgSO_4_·7H_2_O, 3.4 g KH_2_PO4, 2% glycerol, pH adjusted to 7 with KOH) (49) liquid medium for 48 h. The resulting bacterial culture was diluted and plated on BG agar medium, and cultured at 28°C or 37°C. Colonies were randomly selected for gene deletion assay *via* PCR using primers SF + SR listed in Table S3.

Colony samples of *R. solanacearum* recovered from infected tomato plants were subjected to whole-genome resequencing *via* an Illumina NovaSeq 6000 platform at Annoroad Gene Technology, Beijing, China. The sequencing clean reads (deposited at GenBank, BioProject ID PRJNA1012353) were assembled *via* SPAdes (50). The *hrpB* locus and its flanking sequence were used as query and aligned to the resulting scaffolds from genome assembly *via* BLASTn. The 57.4 kb DNA deletion in colonies S6, S7, S15, and S20 was verified *via* PCR using primers 6F + 6R listed in Table S3. The 269.3 kb DNA deletion in colony S8 was verified *via* PCR using primers 8F + 8R, which are listed in Table S3.

### Pathogenicity phenotyping

The pathogenicity assays were conducted following the previously described method (51). In brief, the susceptible tomato cultivar Zhongshu No. 4 or tobacco Yunyan87 was cultured in a greenhouse for 4 weeks and used as the test host plant. *R. solanacearum* was cultured in BG medium with the infecting of the engineered phages or the parent phage RSCq for 12 h. *R. solanacearum* without phage infection was used as a control. The resulting culture was adjusted to 10^7^ CFU/mL and injected into the stems of 32 tomato plants or 15 tobacco plants. The wilting symptoms of the inoculated plants were scored on a visual scale of 0 (no symptoms) to 4 (complete wilting) daily. Kaplan–Meier survival analysis was performed with the Gehan-Breslow-Wilcoxon method to assay the effect of the engineered phage infection on virulence. Three times of pathogenicity assays were performed, and one representative result was presented.

### Infectivity assay of engineered phage in soil

*R. solanacearum* GMI1000 cultured in BG medium was centrifuged and resuspended in sterile H_2_O at a final concentration of 10^9^ CFU/mL. The resulting culture was mixed with sterile soil substrate at a final concentration of 10^8^ CFU/g substrate. The soil that contains pathogens was treated with the engineered phage RSCqYFP01 at the same volume as GMI1000 one day after GMI1000 treatment. The resulting soil was sampled and spread-plated on BG agar medium. The kanamycin resistance of colonies was assayed by replica plating. The phage infection rate was represented by the rate of kanamycin-resistant colonies. This experiment was performed three times independently.

### Biocontrol assay of engineered phages

For the biocontrol assay, *R. solanacearum* strain Tb04 cultured in BG medium was centrifuged and resuspended in sterile H_2_O at a final concentration of 10^9^ CFU/mL. The resulting culture was mixed with soil substrate at a final concentration of 10^8^ CFU/g substrate. The engineered phages RSCqCRISPR-Cas, RSCqYFP01, and the parent phage RSCq were obtained by centrifugation and filtered through a 0.22-µm membrane from the culture supernatant of infected *R. solanacearum*. The soil that contains pathogens was treated with the phages RSCqCRISPR-Cas, RSCqYFP01, or RSCq infection at the same volume with Tb04 1 day after Tb04 watering. The soil without phage treatment was set as a control group. Three-week-old tobacco Yunyan87 seedlings were transplanted into the treated soil 8 days post-phage treatment. In all, 15 tobacco plants were transplanted for each experimental group. The wilting symptoms of tobacco plants were scored. The control efficiency of phage treatment on bacterial wilt (%) was calculated as (disease index of plants in nontreated soil − disease index of plants in phages-treated soil)/disease index of plants in nontreated soil ×100. Kaplan–Meier survival analysis was performed with the Gehan–Breslow–Wilcoxon method to assay the effect of engineered phage treatment on plant bacterial wilt. Three times of biocontrol assays were performed, and one representative result was presented.

## SUPPLEMENTAL MATERIAL

Text S1 mbio.00619-24-s0001.docx Whole genome sequence of the engineered phage RSCqYFP01.

Text S2 mbio.00619-24-s0002.docx Whole genome sequence of the engineered phage RSCqCRISPR-Cas.

Figure S1 mbio.00619-24-s0003.docx Replicative-form DNA map of the engineered phage RSCqYFP01.

Figure S2 mbio.00619-24-s0004.docx Luminescence imaging of *R. solanacearum* GMI1000 infected with engineered filamentous phage RSCqluxA or RSCqluxB or coinfected with RSCqluxA/RSCqluxB, spread plated on BG medium.

Figure S3 mbio.00619-24-s0005.docx Replicative-form DNA map of the engineered phage RSCqCRISPR-Cas.

Figure S4 mbio.00619-24-s0006.docx Virulence assay of engineered-phage-infected *R. solanacearum* Tb04 on tobacco Yunyan87.

Table S1 mbio.00619-24-s0007.xlsx Prophage in 74 *R. solanacearum* species complex strains with publicly available completed genome.

Table S2 mbio.00619-24-s0008.xlsx Tested *R. solanacearum* strains used for the host range determination of the engineered phage RSCqYFP01.

Table S3 mbio.00619-24-s0009.xlsx Primers used in this study.

ASM does not own the copyrights to Supplemental Material that may be linked to, or accessed through, an article. The authors have granted ASM a non-exclusive, world-wide license to publish the Supplemental Material files. Please contact the corresponding author directly for reuse.
