# Supplementary material for: Trojan Horse virus delivering CRISPR-AsCas12f1 controls plant bacterial wilt caused by Ralstonia solanacearum
Source: mBio. 2024 Jul 16;15(8):e00619-24. doi: 10.1128/mbio.00619-24 (PMC11323561; doi:10.1128/mbio.00619-24)
Supplement: Text S1 — Whole genome sequence of the engineered phage RSCqYFP01. [file mbio.00619-24-s0001.docx]

**Supplementary Text 1****.** The whole genome sequence of the engineered phage RSCqYFP01

cgtttacccctcgcccgggttaaccggctatatttacgccagaatcacgggcgtaattcatcacggacataatattaattacatccttcatgccgtcaagcaaagaggtgcttagatgagaattgagaaatacttagatcaggcgatcgaacgccacggcctgaagaacgacagcaagctggcagagatgctaggtgtggtgcaaagcgcggtcagccactaccgcaccggccgccgcacggcggacaacgaagtgtgcctccgcctggcgcagctgctcgagatggagaacccgctgccgatcatcatggcggccgacatggaccgcgccgaacgtgctggccagcactctctctgggaagttttttcgacgaggatggcagccagtaacgcgacagccgccctcctcctggtactggtcgcgagcgcaacaaattttgttgcgccctctcccgccaaagccgcgccgttgagccattcgacagctcaacgattattgttatgtaaaatagctcgccgacttcgggagcgcctacagcaagcgctgcgtgcactgtctcttatacacatctcgcgcaacgcaattaatgtgagttagctcactcattaggcaccccaggctttacactttatgcttccggctcgtatgttgtgtggaattgtgagcggataacaatttcacacaggaaacacatatgatggtgagcaagggcgaggagctgttcaccggggtggtgcccatcctggtcgagctggacggcgacgtaaacggccacaagttcagcgtgtccggcgagggcgagggcgatgccacctacggcaagctgaccctgaagttcatctgcaccaccggcaagctgcccgtgccctggcccaccctcgtgaccaccttcggctacggcctgcagtgcttcgcccgctaccccgaccacatgaagcagcacgacttcttcaagtccgccatgcccgaaggctacgtccaggagcgcaccatcttcttcaaggacgacggcaactacaagacccgcgccgaggtgaagttcgagggcgacaccctggtgaaccgcatcgagctgaagggcatcgacttcaaggaggacggcaacatcctggggcacaagctggagtacaactacaacagccacaacgtctatatcatggccgacaagcagaagaacggcatcaaggtgaacttcaagatccgccacaacatcgaggacggcagcgtgcagctcgccgaccactaccagcagaacacccccatcggcgacggccccgtgctgctgcccgacaaccactacctgagctaccagtccgccctgagcaaagaccccaacgagaagcgcgatcacatggtcctgctggagttcgtgaccgccgccgggatcactctcggcatggacgagctgtacaagtaatctagaccgccacggttgatgagagctttgttgtaggtggaccagttggtgattttgaacttttgctttgccacggaacggtctgcgttgtcgggaagatgcgtgatctgatccttcaactcagcaaaagttcgatttattcaacaaagccgccgtcccgtcaagtcagcgtaatgctctgccagtgttacaaccaattaaccaattctgattagaaaaactcatcgagcatcaaatgaaactgcaatttattcatatcaggattatcaataccatatttttgaaaaagccgtttctgtaatgaaggagaaaactcaccgaggcagttccataggatggcaagatcctggtatcggtctgcgattccgactcgtccaacatcaatacaacctattaatttcccctcgtcaaaaataaggttatcaagtgagaaatcaccatgagtgacgactgaatccggtgagaatggcaaaagtttatgcatttctttccagacttgttcaacaggccagccattacgctcgtcatcaaaatcactcgcatcaaccaaaccgttattcattcgtgattgcgcctgagcgagacgaaatacgcgatcgctgttaaaaggacaattacaaacaggaatcgaatgcaaccggcgcaggaacactgccagcgcatcaacaatattttcacctgaatcaggatattcttctaatacctggaatgctgtttttccggggatcgcagtggtgagtaaccatgcatcatcaggagtacggataaaatgcttgatggtcggaagaggcataaattccgtcagccagtttagtctgaccatctcatctgtaacatcattggcaacgctacctttgccatgtttcagaaacaactctggcgcatcgggcttcccatacaatcgatagattgtcgcacctgattgcccgacattatcgcgagcccatttatacccatataaatcagcatccatgttggaatttaatcgcggcctcgagcaagacgtttcccgttgaatatggctcataacaccccttgtattactgtttatgtaagcagacagttttattgttcatgatgatatatttttatcttgtgcaatgtaacatcagagattttgagacaccctgtgaatgcgcaaaccaacccttggcagaacatatccatcgcgtccgccatctccagcagccgcacgcggcgcatctcgggcagcgttgggtcctggccacgggtgcgcatgatcgtgctcctgtcgttgaggacccggctaggctggcggggttgccttactggttagcagaatgaatcaccgatacgcgagcgaacgtgaagcgactgctgctgcaaaacgtctgcgacctgagcaacaacatgaatggtcttcggtttccgtgtttcgtaaagtctggaaacgcggaagtcagcgccctgcaccattatgttccggatctatgtcgggtgcggagaaagaggtaatgaaatggcagatccctggcttgttgtccacaaccgttaaaccttaaaagctttaaaagccttatatattcttttttttcttataaaacttaaaaccttagaggctatttaagttgctgatttatattaattttattgttcaaacatgagagcttagtacgtgaaacatgagagcttagtacgttagccatgagagcttagtacgttagccatgagggtttagttcgttaaacatgagagcttagtacgttaaacatgagagcttagtacgtgaaacatgagagcttagtacgtactatcaacaggttgaactgctgatcttcggatctatgtcgggtgcggagaaagaggtaatgaaatggcatccggatctgcatcgcaggatgctgctggctaccctgtggaacacctacatctgtattaacgaagcaattcatcgatgatggttgagatgtgtataagagacaggtccaaacaagcccgaaaacggcaccgtgctgatcgacagcacagcagcagatctagcacctcccgttccgaaacgcctacagcaagcgctgcgtgcagtccaaacaagcccgaaaacggcaccgtgctgatcgacagcacagcagcagatctagcacctcccgttccgaaacgcctgcatctagcaggcgtttttttttcgtgcggatctttcgatagtaccgatgccgagcggcatcctggtagccccggcgcagtgtccggatatgccagtacagatgcctgatctcgcgcgccacggccttcagttcgtcgtctgacactgccgattttggttgttctgagcagcgtagcatcggcacgcgaagcgatccgccacagccagcgcaaccgaagatcgtaagcgtttcgaaagctggcttgcaggaaagggggtgtttgtatgtgggcggccctgtatgtacgtacctggtcatacccgctgctcggtcccgccaggcgccctccgggcatcaaccgtcctgcacctggtcaactctcccatgtccccagaggggccccctagcggctccgactacggcgcggacactcccgacgctggacgcaaaaaaaggccggcgatccgcacggcctttctatttttcgttaccttttcgttactccgggtagaactccggcgacagcacctgctcgggtatccacggccactgctcggggaacacgtccaggccagtctcgtccacagccgccgagaccgcatccgcccagatctcttccttccaatccggatcagccaacatcggctgcaggctgggcgtccgatgcaaccgcgccagaattgcgcgacgctgttccttgatcgtgcgctgccagctcgaaccccggcggcccggctgatattgccacttgagcaaatgcgccagcagtaccgccatgcggcttgccaactcccgctgttcgctcttgcccacgtcctcaatctcctccgcgatgtgccgaatgtcgatgtctgaaagcttgccggcccgcaacagcgccgcctgctcactggcccacgccaccacgtccacttcgtagctcgttcccataacgcgctccctccaataaccggatcattttagacgtttcgcgttacgccttgcgcgtaacgactttgcgatcaccataaattgcctcgccagccgcttgcatctgcgcctcactcacggccacagaacggacaaacgaagcgcgccacaactcggactcccgcaccttctggcgctgttcgaacgtattcgccttcgcagccaacgcgagcaagtccagcattgcgtcgaacgaaacgggatttccgttactcggcgtaacggatttttgctgcgactcaaacatcgcaaccttctcttccaagacgccaacataacgcaacgcttcctgacgcccgtcctcgcacgtcataagctgccggcgcaacgcctcaatctccgcaagatttccgttactcagcgtaacggatttggcggcatcgctgatctcttccctcagccgcctattctcagcaaacgccttattgcgttcagcctccgccagctccacctggcgacgcatgctcaccagatcagcgcgaaacttctcgacctccaggcggcattcatcgtaagcatccacgtcggaaagcatttccgttacggtcatacgcttagtaacggatttagcctcaacaggctgctgagccttacggcgagctcgatatgcggcttgacgctccgcgttggtcatagcgtgcgccttacgcggacgaccgcggccacgcctttgctccatccccggcagatcgaccgtcacattatccgttacgtcacgcatattcggctcccgatttgatgattccattttacgttactggtaacggcaattcaattatcgttacaatttagttcgtaacggaaatttcgcgagcagccccggcgctagacgccctcagcgggctttggagccgttttttctgactgggcggtcgcaggggtcagctgcgccgaaattggctcctgcgccgcttgtggctcgcggtaggggtcaaacggcggcctcctgatccactcccgacactgcatgtcatcgaggccggcatccgtgccttgagccgtgtagcacgtgcacctggtcgacgtgcaggcaccgccgatcaccgtcggcatcgagcgaatctggcgcaactgcgcataggccggcgccgtctcaggccggccagagacagcagggacgaacgccgcgaggatctccgccccgtccgtcgccgcggcggacggacccgccttgaccgctaccgctagagctgaagccacaccgcccctgccctgctcggccgcaacaggcttagccggagtcgccgtcgtcaactggccggtacggtaatagacccggtacgccaagaacgccgcgatcaacacgcaggcgatgaacagcatcagcaccggcggtacggtgtacttgcgcttgatgtgcagactggaggacttgtacaggccgaaactggacttcggcaggctccacttcttcttgatcggcgcggtgttgaacgtctccgggttcgcgcactccggccattcgtagtaccagcgtccgagcaagccagcgtcccgcaggtggacatgctgccccaccagcttgcggatatgactgtccaagaacgtcgggttctgcgtgatcagaacaaacgtcacgccggtatgccgcaccgtctcaaacgccgccacgtggtcaggcaccttggacccggccgtgcggacgcgaaacacccgctgcgcctcatccaacacgatcagcgagttcggcgggaaggtgaagtacggcagcatcatccctgggttttcagggtcttcgcgcagctccgtccagtccgaaaccgccggcgtcgggatatacggcagcttcagctccgggatgcccatgacgaagagcggacgcccctggtcgacggctgccttcatcatctggaccgccaacgcggtcttcccgccaccaggcgtggccgtgatcagcgtgatcggttgcgttgcgctcatgtcagcttgcccagccgcttcagagtgatcatggagatgcgcgcggtgatgccgccggcgatgatcgacagcccggtgaagacaccgccccgcgccaggatggccgcagcaacggcaggcatgccggccaggctactcttggccgcgcccagggcagcgctcaccgccgcatccaacccgacataggtgatcagaccgatcccgagcgacaccagtagctgacgcgcgagtggcccaacgagggccatgaggaacccagcgagcggcatcactcctcccctttacggcctacgccgatgacgatcagcgcagcgcccaaccatgcgcacgcgatgatgaccggtcgaaacatgtcagcaccgtcacagaccggtttgagcgaccaagagatcggcatgccgtggatggaggcggtcagatcggacggacacggtgcggtatctgcgccccaaccgctgtccggcatgaccttgacgttgacctgctgctccttcagatcgggcccgtccggtatctcgccttgctcgatacaacccatgcgcgtctcatggcccgagcactggtcgtcctgccgctccggagccttgccgttaccagtcgccggatccgtcgccggattgccgttcgcatccacctcctgcttagcggtcgtcaacgtggctgtcttgccatcggaatttggcgtgaccgtagcaacatcgcggtaacgcttacctgtaacgggatcaacgtacgggtcgctcagattgacgttgactggagtggtggacggcgtgagcttcaccggaatcggcaccttggctgcggccatatcgctagcaacagcagcgggcagcggatacgtcaaacctttgttccaatccgcatcgcttgcgcccaccgtcggaccggcagggtcgggcacacatgccgagccactcacgacatagccatcaacgcagctggacgcctgactcgtgcccgcatagaaattgtcgccgccatcgttggtgtaatggcactcatagctcgtcccattaccggtagctttcatacccgcgaacttagccttctgcccagccaaatacgcgtctgccgccaacatcgcagagcacgcggcgccaggagaagcagcaacgccattcgcgatattgccgccggtggcggacgtgttgtagccgtaattccagccccagccattgaaccccgtgtcgccagcagccggtgaccgcttcgatgtgcaccacgtaccgtccaggcacttctggatgccgagctgcgccaagtaggccaacgacgtcgcggtcgcgatcgcaggcgtcgcacgaagcgcggccagcgcaacggcagcagcgccttcgcccaccgtcatcgttgcggcgatcgtggcggcacctgcgccaaccgcggcacgcatctcggtagccgtcgcaacttcagacagcgtcacagcaccagtcgtgacatacccagtccccgtcaacacgatgttcggcggcggaatcaacgggatggtcgaagcccacgcggacgacgcccaccagcagagcagcagcacgagcacgcgcatcacagccccctgaaaatgattactgccggcagaaccggcagcaggaatccggcccagagccagaaatcgatcgcgagcatcacagcccctttttcatcaccaccagcgcccaggccgccaccatcgcggcaacgacgccccaccccatcgtcatgccatccttgaagctctcttgcgggtcacatgccggaaacgacagcgacggcaacgacgcatcggtcaacgtgccgacgctcccatcactgcccacctgataccgccgcagcacccacccgcccgtggtcttgacgaactcagacagataggtgactgccccaggtgtctgcgacggagcgacggcgctgtaataggcatccgtggccatgcccacatcggcaaagcaccgtgcgcccaccaacgcgccgtcagcagccatcacacgctccgacgcatgtacttgaacgtcgcaatgccgatcacgatgatcagcgccaggcccgccagcgtggcggtgtccgccttcgaatcggacatggcggtagcaacgtcggtcggcaccgcagccatcgccgaaccggccagtgcaacggtacccgcggcaacagcagccgccttgctcttgatgctcttgaacatggttttctccttgagatggaggttgagaaaagctccgggccgttcaactcccagagccagggaacatcacgccttcttgggctcagcctcagcacgctgcagcggcttgatgctcgtaacgaccttctgaccgcccttatccttgccgttgctcgtctcgaccatcgagacttccgcgatgaacgggaacgggttgtggatgatcgccttcacaaccgcagagctctcgcacttcagctcctgcgtgcaggtgcccttcgagtcttcgccacgcagctccacatccgtgtagatcttcccggtgtccagctgcttgccatccatgttgccaacccacgtcttggcgccccggatggtcacgcgtgcaatcatttccatggtttcactcctcaggtttcggcactggatcgtgcgtgccgtacacgtgcctcgccagggcggatttgtgcagctgagccggtacgccctgacggcgtatcgcgacaaccagcgcagcgatgtcttcctctgtgcagcgcagctcgtaatcgaccgtagggccgaactgcgtctggatgtgcttgagcttgcgttcgcggatggtctcgtcctgaagctccaaggccttgacctggtcagtaggaacgcgctgcggatccgcagccatgaaggcctccagggccttgtacgcaccagcgaagtactggtcgcgcttgataaggatttcgtgcggaatcacgcgatccttggcgccgaactcgatctcgagacgcacccactcgctatcctgattgccgagctggcggcccttctcgtaagcccgcagcatcttgccgttcgcccgacggccaatctcgaacgtcgtaccgcgacagcccttgctgcccgccacgccgctctcgatcttgcgatacgtcgggatacgcccgcccgcgttgaagtcgccggcgtagtacagctcttccatctgcgcgatgctcacctcgccctggcagaagtccatcgccaggtcgcaccgcgtgatccgcgcgtcgaggtcctgcaccatcgcgtagacggcttgccagtcgccaatcgcggtgcagccctgccccggccagtccaccaggatcgtgccgccaacgtgctcgccgccgcaggcaacgatcccgagcttcatcgtctcgccgttgatgaacgccagcaggtcgtagctgaactcataccggcggaaccctttgcccgcaggcttcatcgtcaccggcaccgagaacaccagctggaagtacctgcgcagctgctccagggcgtcgctgatgctgccgtcgggcaggaacgtgaacttgaaccagtccacgatcgcacctgccttgcgttctggactttccccgggttttaccggacccggggaacggcctgtcggccgcccagcctcgctccgctcggcccggtcggccgccatgccgttctcgcgtgcgagcgccggcccatgaactccatacgcaatgcctgtgggatcaagatgacgagagggggtgggacggacgcgcatcagcgtgcgccctcgcagcgataccagttcgccagatcggcctggcgcgagttcttcaggaggtcgtcaatgcgatgcatcgcaggacgcgattgcaggcagcaggcgccgcgcagaacggcagccatcacgatggccagctgcgcaccgtgcgggtgaagcacgtcgccaatcaggtccagctgcctatggtctctctgctgcat
