# Supplementary figures and images for: Trojan Horse virus delivering CRISPR-AsCas12f1 controls plant bacterial wilt caused by Ralstonia solanacearum
Source: mBio. 2024 Jul 16;15(8):e00619-24. doi: 10.1128/mbio.00619-24 (PMC11323561; doi:10.1128/mbio.00619-24)

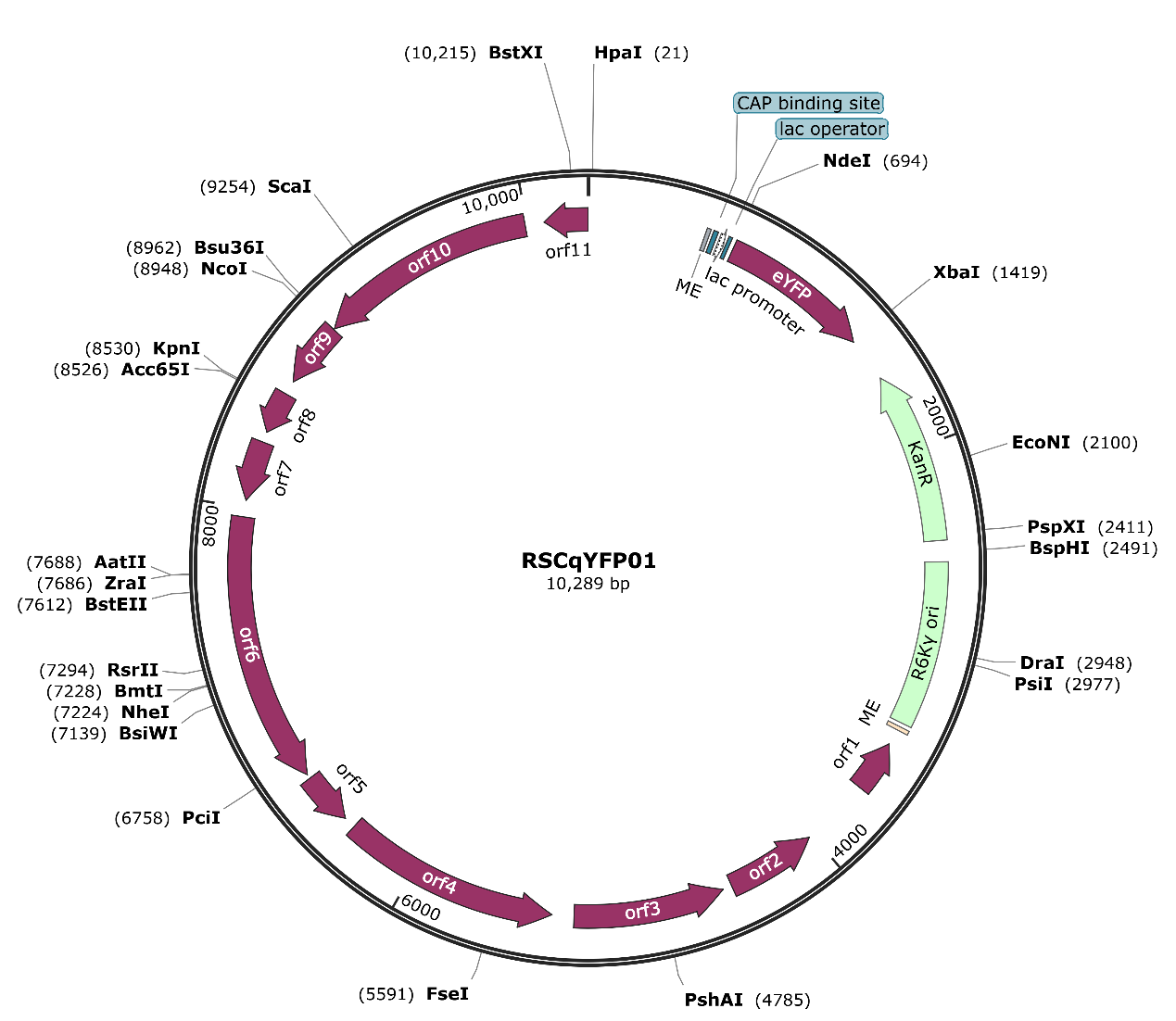


**Figure S1**. The replicative form DNA map of the engineered phage RSCqYFP01

Supplement: Figure S1 — Replicative-form DNA map of the engineered phage RSCqYFP01. [file mbio.00619-24-s0003.docx]

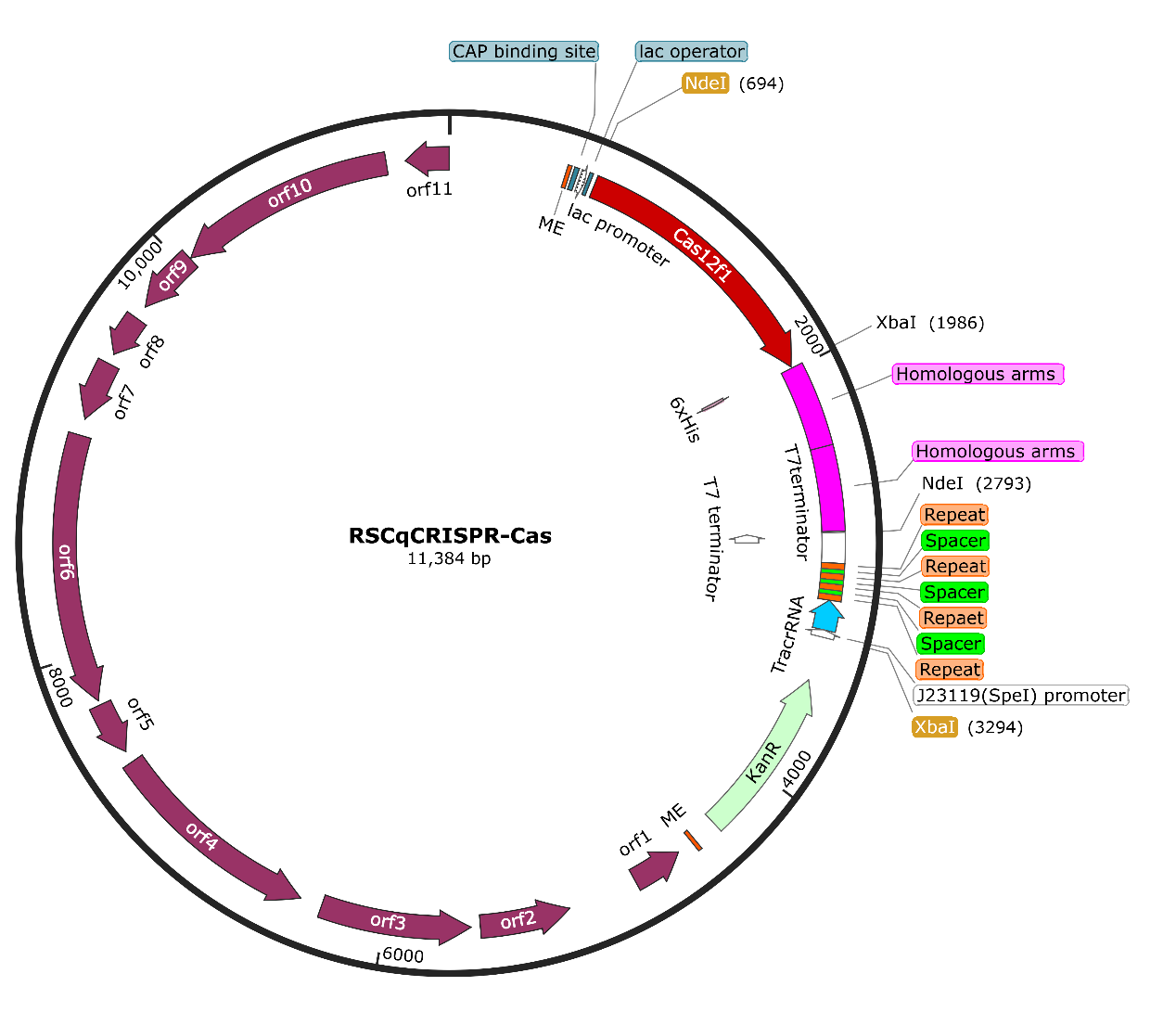


**Figure S3**. The replicative form DNA map of the engineered phage RSCqCRISPR-Cas

Supplement: Figure S3 — Replicative-form DNA map of the engineered phage RSCqCRISPR-Cas. [file mbio.00619-24-s0005.docx]
