# Supplementary material for: Trojan Horse virus delivering CRISPR-AsCas12f1 controls plant bacterial wilt caused by Ralstonia solanacearum
Source: mBio. 2024 Jul 16;15(8):e00619-24. doi: 10.1128/mbio.00619-24 (PMC11323561; doi:10.1128/mbio.00619-24)
Supplement: Figure S2 — Luminescence imaging of R. solanacearum GMI1000 infected with engineered filamentous phage RSCqluxA or RSCqluxB or coinfected with RSCqluxA/RSCqluxB, spread plated on BG medium. [file mbio.00619-24-s0004.docx]

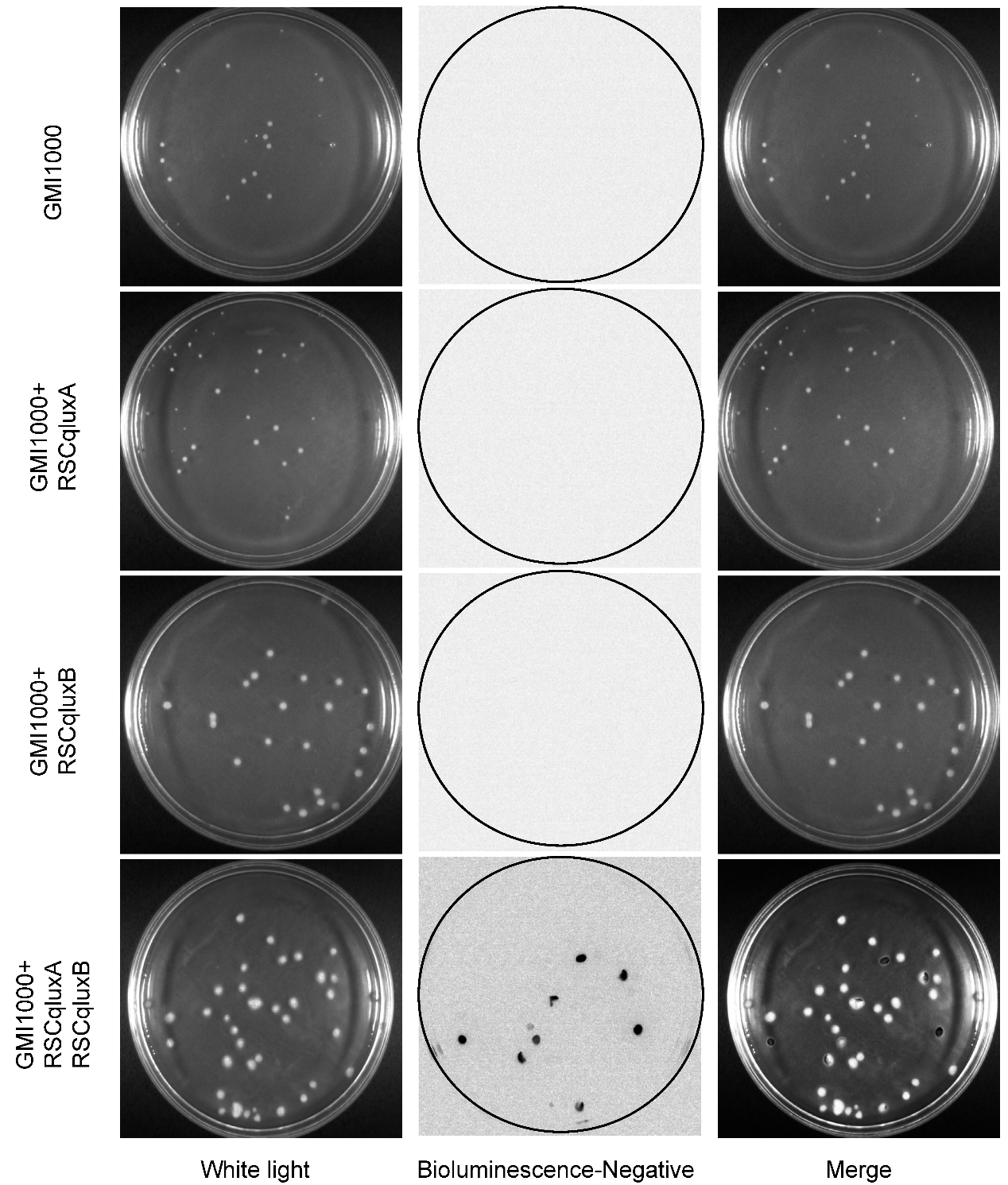


**Figure S2**. Luminescence imaging of the engineered filamentous phages RSCqluxA- or RSCqluxB- infected, or RSCqluxA/RSCqluxB co-infected *R. solanacearum* GMI1000 spread plated on BG medium. Left panel, image of RSCqluxA/GMI1000 and RSCqluxB/GMI1000 under white light. Middle panel, the negative image of bioluminescence in the dark. Right panel, merged image.
