# Supplementary material for: Trojan Horse virus delivering CRISPR-AsCas12f1 controls plant bacterial wilt caused by Ralstonia solanacearum
Source: mBio. 2024 Jul 16;15(8):e00619-24. doi: 10.1128/mbio.00619-24 (PMC11323561; doi:10.1128/mbio.00619-24)
Supplement: Figure S4 — Virulence assay of engineered-phage-infected R. solanacearum Tb04 on tobacco Yunyan87. [file mbio.00619-24-s0006.docx]

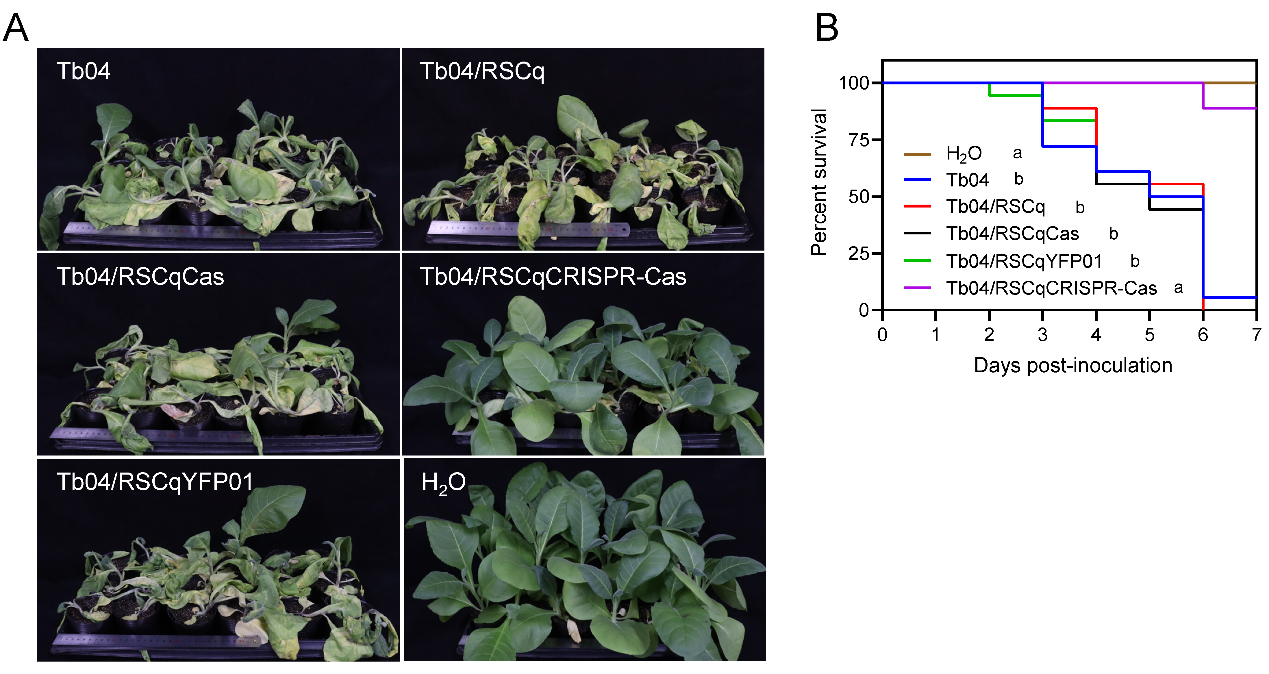


**Figure S4**. Virulence assay of engineered phages infected *R. solanacearum* Tb04 on tobacco Yunyan87. **A**. Bacterial wilt symptoms of tobacco plants seven days after inoculation with *R. solanacearum* Tb04, Tb04 infected with phage RSCq or engineered phages. **B**. Survival curve of infected tomato plants. Kaplan-Meier survival analysis with the Gehan-Breslow-Wilcoxon method was used to compare pathogenicity between the mutant and wild-type strains. Different letters represent significant differences at P < 0.05 probability level.
